# Leg length and sitting height reference data and charts for children in the United States

**DOI:** 10.1016/j.dib.2020.106131

**Published:** 2020-08-05

**Authors:** Colin Patrick Hawkes, Sogol Mostoufi-Moab, Shana E McCormack, Adda Grimberg, Babette S. Zemel

**Affiliations:** a Division of Endocrinology and Diabetes, The Children's Hospital of Philadelphia, Philadelphia, USA; b Department of Pediatrics, The University of Pennsylvania Perelman School of Medicine, Philadelphia, USA; c Division of Gastroenterology, Hepatology and Nutrition, The Children's Hospital of Philadelphia, Philadelphia, USA

**Keywords:** Height, Sitting height, Skeletal dysplasia, Reference, Data

## Abstract

Population-specific reference data are required to interpret growth measurements in children. Sitting height and leg length (standing height minus sitting height) measurements are indicators of proportionality and can be used to evaluate children with disordered growth. NHANES III recorded sitting height and standing height measurements in a strategic random sample of the United States population from 1988 to 1994, and we have previously published reference charts for sitting height to standing height ratio in this population. In this study, we have developed separate sitting height and leg length reference charts for Non-Hispanic Black, Non-Hispanic White, and Mexican-American children in the United States. In addition, we provide mean (SD) and LMS data to support the use of these reference charts in clinical care.

**Data Specifications table**

**Table:** 

Subject	Endocrinology, Diabetes and Metabolism
Specific subject area	Sitting height and leg length reference data for children in the United States
Type of data	Growth Charts and Tables
How data were acquired	NHANES III Data Files https://www.cdc.gov/nchs/nhanes/nh3data.htm
Data format	This is a secondary analysis of data from the NHANES III survey.
Parameters for data collection	Anthropometric measurements and self-reported ethnicity from the NHANES III survey and were used to generate these reference data.
Description of data collection	The NHANES III survey randomly selected subjects from households in 81 counties in the United States.
Data source location	Primary data source: NHANES III Survey, Examination File
Data accessibility	Raw data can be retrieved from NHANES III Series 11 Data Files https://wwwn.cdc.gov/nchs/data/nhanes3/1a/exam.dat
Related research article	Hawkes CP, Mostoufi-Moab S, McCormack SE, Grimberg A, Zemel BS. Sitting height to standing height ratio reference charts for children in the United States. J Pediatrics 2020. DOI 10.1016/j.jpeds.2020.06.051.

**Value of the Data**

• These data provide previously unavailable reference charts for leg length and sitting height for children in the United States.
• The charts presented here will allow clinicians to assess whether a patient's sitting height or leg length is within the range expected for their ancestry, sex, and age. Clinically, this will be useful in identifying children with disproportionate growth and in monitoring proportionality in children at risk for attenuated spinal growth (e.g. following craniospinal radiation treatment).
• These data will also allow clinicians and researchers to determine percentiles and Z-scores for sitting height and leg length in children, and facilitate longitudinal monitoring of these measurements over time.

## 1 Data Description

The National Health and Nutritional Examination Survey (NHANES) is performed periodically in the United States and aims to enroll a representative sample of the United States population [1,2]. NHANES III, performed in 1988–1994, is the last survey to include sitting height measurement in children. Standing and sitting-height measurements performed during this survey were used to generate these data.

For standing height measurements, the subject was required to stand on the floor-board of the stadiometer with both heels together. His or her heels, buttocks and scapulae were touching the vertical backboard and the subject's arms were allowed to hang freely by their side, with palms facing thighs. When sitting height was measured, the subject was seated on a measurement box with their back and buttocks touching the backboard of the stadiometer, knees directed straight ahead, arms and hands resting at their side. For both standing and sitting height measurement, the head was in the Frankfort Horizontal Plane [3]. Leg length was calculated by subtracting sitting height from standing height.

**Fig. 1:**
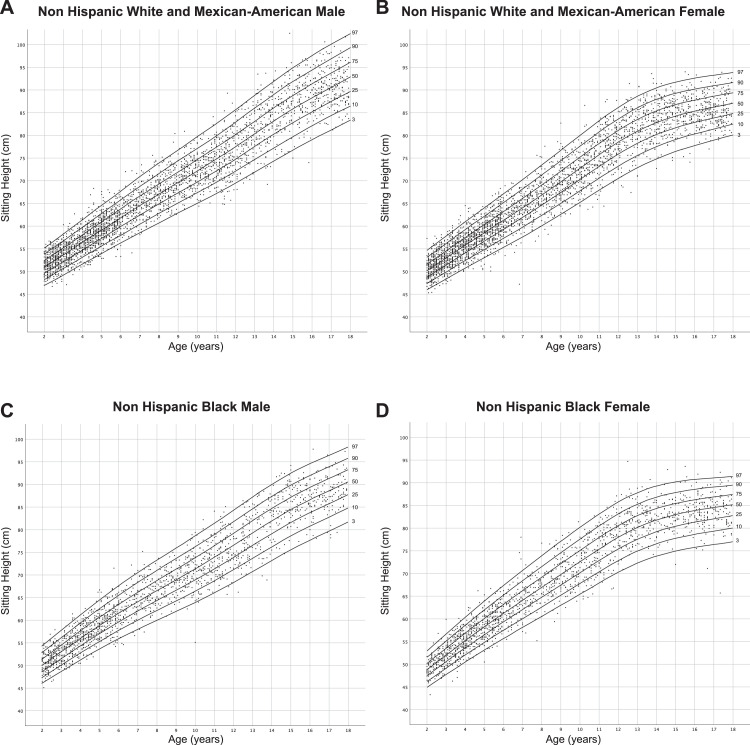
Ancestry and sex-specific sitting height reference charts for Non-Hispanic White, Non-Hispanic Black and Mexican-American youth in the United States.

**Fig. 2:**
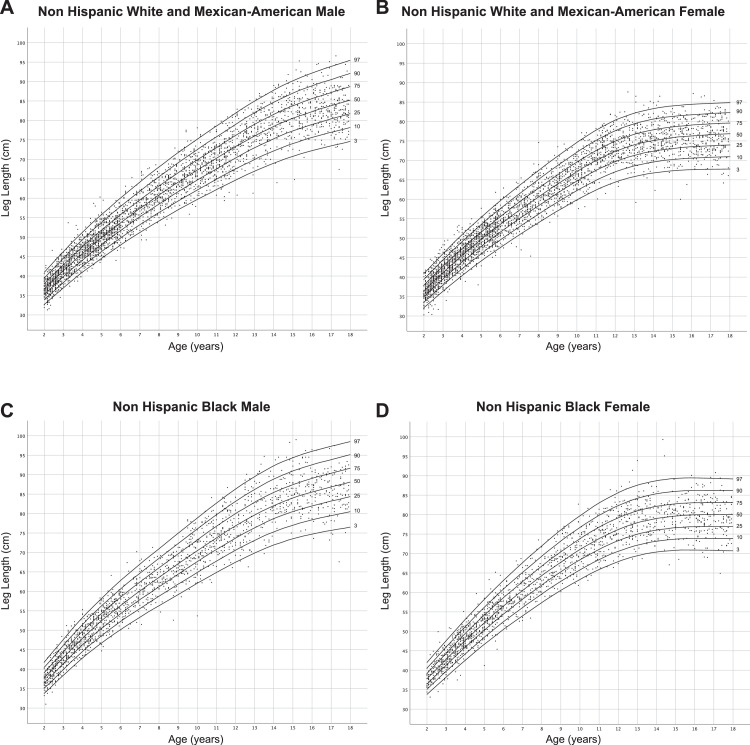
Ancestry and sex-specific leg length reference charts for Non-Hispanic White, Non-Hispanic Black and Mexican-American youth in the United States.

Figs. 1 and 2 show sex- and ancestry-specific centile charts for sitting height and leg length respectively and can be used to track growth of these parameters over time. Tables 1 and 2 provide sex- and ancestry-specific mean (standard deviation) values for sitting height and leg length in this population, respectively. This provides an additional tool for interpreting these measurements in children. Table 3, Table 4, Table 5, Table 6 provide L, M, S, and percentile values for sitting height while Tables 7–10 provide these data for leg length, and will support integrating these growth charts to electronic health records or calculating an individual child's Z-score for each measurement.

## 2 Experimental design, materials and methods

Sitting height and standing height were measured and leg length was calculated for each subject with available measurements aged 2–18 years. In order to adjust the data from study participants to reflect the United States population, weighting was assigned to each subject (designated WTPFEX6 in the NHANES dataset [4]). This weighting was used to generate population-specific reference charts and all analyses, as described elsewhere [5].

Non-Hispanic black (NHB) children in the United States were found to have differences in body proportions when compared with non-Hispanic white (NHW) and Mexican-American children [6]. To account for these differences, separate sex- and ancestry-specific reference charts for NHB and NHW/Mexican American male and female children were generated.

LMS Chartmaker Pro (Harlow Printing Ltd., Tyne and Wear, UK) was used to generate sitting height and leg length reference charts. The LMS method [7] uses a Box-Cox transformation to obtain normality, and separate smooth curves are generated for skewness, median and variability. These are constrained to smooth changes over time and combined in one graph. As per software recommendations, these were adjusted until the fit of the curve was visually optimized. LMS files were generated for each chart.

## Ethics statement

For participation in the NHANES III survey, parental consent was provided for all children under 18 years of age and the protocol was approved by the National Center for Health Statistics Research Ethics Review Board.

**Table 1:** Mean (SD) sitting height in non-Hispanic white / Mexican American or in non-Hispanic black children.

Age, years	Non-Hispanic White or Mexican-American				Non-Hispanic Black			
	Male		Female		Male		Female	
	Mean	SD	Mean	SD	Mean	SD	Mean	SD
2	51.02	2.04	50.20	2.16	50.05	2.05	48.87	2.00
2.5	52.37	2.09	51.61	2.26	51.57	2.16	50.50	2.12
3	53.73	2.15	53.03	2.35	53.07	2.26	52.11	2.23
3.5	55.09	2.20	54.43	2.45	54.57	2.37	53.72	2.35
4	56.46	2.26	55.83	2.55	56.06	2.48	55.30	2.46
4.5	57.83	2.31	57.20	2.65	57.51	2.59	56.86	2.58
5	59.20	2.37	58.57	2.75	58.93	2.70	58.38	2.70
5.5	60.57	3.03	59.94	2.85	60.30	2.81	59.86	2.81
6	61.93	3.10	61.31	2.95	61.64	2.92	61.33	2.93
6.5	63.28	3.16	62.69	3.05	62.94	3.02	62.77	3.04
7	64.62	3.23	64.09	3.15	64.21	3.12	64.20	3.15
7.5	65.93	3.30	65.50	3.24	65.45	3.22	65.62	3.25
8	67.23	3.36	66.94	3.34	66.65	3.31	67.04	3.35
8.5	68.51	3.43	68.39	3.43	67.85	3.41	68.46	3.45
9	69.77	3.49	69.87	3.52	69.04	3.50	69.88	3.53
9.5	71.03	3.55	71.36	3.60	70.25	3.59	71.30	3.61
10	72.30	3.62	72.86	3.68	71.48	3.67	72.72	3.69
10.5	73.59	3.68	74.36	3.75	72.73	3.76	74.13	3.75
11	74.91	3.75	75.83	3.81	74.03	3.84	75.50	3.80
11.5	76.27	3.81	77.25	3.86	75.36	3.92	76.83	3.84
12	77.67	3.88	78.59	3.89	76.71	3.99	78.08	3.87
12.5	79.09	3.95	79.83	3.91	78.09	4.05	79.21	3.88
13	80.53	4.03	80.95	3.92	79.47	4.10	80.22	3.88
13.5	81.96	4.10	81.95	3.91	80.83	4.14	81.11	3.87
14	83.36	4.17	82.83	3.88	82.15	4.18	81.86	3.85
14.5	84.73	4.24	83.58	3.85	83.41	4.20	82.50	3.82
15	86.05	4.30	84.24	3.80	84.59	4.20	83.05	3.78
15.5	87.31	4.37	84.81	3.75	85.71	4.20	83.51	3.74
16	88.53	4.43	85.32	3.68	86.76	4.19	83.91	3.69
16.5	89.71	4.49	85.78	3.62	87.76	4.17	84.26	3.64
17	90.86	4.54	86.21	3.55	88.74	4.15	84.58	3.59
17.5	91.99	4.60	86.64	3.48	89.71	4.12	84.89	3.55
18	92.93	4.65	87.00	3.43	90.52	4.10	85.15	3.50

**Table 2:** Mean (SD) leg length in non-Hispanic white / Mexican American and in non-Hispanic black children.

Age, years	Non-Hispanic White or Mexican-American				Non-Hispanic Black			
	Male		Female		Male		Female	
	Mean	SD	Mean	SD	Mean	SD	Mean	SD
2	36.65	2.07	36.48	2.18	37.59	2.01	37.86	2.04
2.5	39.03	2.20	38.86	2.30	40.28	2.16	40.38	2.18
3	41.39	2.33	41.21	2.41	42.93	2.32	42.89	2.33
3.5	43.68	2.46	43.50	2.52	45.49	2.47	45.35	2.47
4	45.89	2.58	45.71	2.63	47.93	2.61	47.76	2.61
4.5	48.00	2.70	47.83	2.72	50.24	2.75	50.09	2.75
5	50.02	2.81	49.85	2.82	52.43	2.89	52.34	2.88
5.5	51.97	2.92	51.81	2.91	54.50	3.03	54.52	3.01
6	53.86	3.03	53.69	3.00	56.46	3.16	56.62	3.14
6.5	55.70	3.14	55.52	3.09	58.34	3.29	58.66	3.27
7	57.50	3.25	57.31	3.18	60.16	3.42	60.63	3.40
7.5	59.25	3.36	59.06	3.27	61.94	3.55	62.54	3.52
8	60.96	3.47	60.79	3.35	63.68	3.68	64.41	3.64
8.5	62.63	3.58	62.48	3.44	65.42	3.82	66.21	3.75
9	64.26	3.69	64.13	3.53	67.15	3.95	67.94	3.87
9.5	65.85	3.79	65.74	3.61	68.87	4.09	69.58	3.97
10	67.40	3.90	67.29	3.69	70.58	4.23	71.14	4.07
10.5	68.93	4.00	68.77	3.77	72.26	4.36	72.59	4.17
11	70.45	4.11	70.13	3.84	73.90	4.49	73.94	4.25
11.5	71.94	4.22	71.35	3.91	75.49	4.62	75.16	4.33
12	73.41	4.32	72.43	3.97	77.01	4.73	76.24	4.40
12.5	74.83	4.42	73.34	4.02	78.46	4.84	77.16	4.45
13	76.19	4.52	74.10	4.06	79.81	4.95	77.93	4.50
13.5	77.47	4.62	74.72	4.09	81.07	5.04	78.54	4.54
14	78.66	4.70	75.21	4.12	82.21	5.11	79.01	4.56
14.5	79.75	4.78	75.60	4.14	83.24	5.18	79.36	4.58
15	80.74	4.86	75.90	4.16	84.16	5.24	79.60	4.59
15.5	81.64	4.93	76.14	4.17	84.98	5.30	79.77	4.60
16	82.47	4.99	76.32	4.18	85.71	5.34	79.87	4.60
16.5	83.24	5.05	76.48	4.19	86.37	5.38	79.93	4.60
17	83.96	5.11	76.62	4.20	87.00	5.42	79.96	4.60
17.5	84.67	5.16	76.75	4.21	87.61	5.46	79.98	4.60
18	85.26	5.21	76.86	4.22	88.12	5.49	79.99	4.60

**Table 3:** Sitting Height in Non-Hispanic White or Mexican American Males.

	LMS Curves			Percentiles						
Age (years)	L	M	S	3rd	10th	25th	50th	75th	90th	97^th^
2	0.46	51.02	0.04	47.00	48.32	49.66	51.02	52.40	53.81	55.23
2.1	0.45	51.29	0.04	47.23	48.57	49.92	51.29	52.69	54.10	55.54
2.2	0.44	51.56	0.04	47.47	48.81	50.18	51.56	52.97	54.40	55.85
2.3	0.44	51.83	0.04	47.70	49.06	50.43	51.83	53.25	54.70	56.16
2.4	0.43	52.10	0.04	47.94	49.30	50.69	52.10	53.54	54.99	56.47
2.5	0.42	52.37	0.04	48.17	49.55	50.95	52.37	53.82	55.29	56.78
2.6	0.42	52.64	0.04	48.40	49.79	51.21	52.64	54.10	55.59	57.09
2.7	0.41	52.92	0.04	48.64	50.04	51.47	52.92	54.39	55.89	57.41
2.8	0.40	53.19	0.04	48.87	50.29	51.72	53.19	54.67	56.18	57.72
2.9	0.40	53.46	0.04	49.11	50.53	51.98	53.46	54.96	56.48	58.03
3	0.39	53.73	0.04	49.34	50.78	52.24	53.73	55.24	56.78	58.35
3.1	0.38	54.00	0.04	49.58	51.02	52.50	54.00	55.53	57.08	58.66
3.2	0.38	54.27	0.04	49.81	51.27	52.76	54.27	55.81	57.38	58.98
3.3	0.37	54.54	0.04	50.05	51.52	53.02	54.54	56.10	57.68	59.29
3.4	0.36	54.82	0.04	50.28	51.76	53.28	54.82	56.39	57.98	59.61
3.5	0.35	55.09	0.04	50.52	52.01	53.54	55.09	56.67	58.28	59.92
3.6	0.35	55.36	0.04	50.75	52.26	53.80	55.36	56.96	58.59	60.24
3.7	0.34	55.64	0.04	50.99	52.51	54.06	55.64	57.25	58.89	60.56
3.8	0.33	55.91	0.04	51.22	52.76	54.32	55.91	57.54	59.19	60.88
3.9	0.33	56.19	0.04	51.46	53.00	54.58	56.19	57.82	59.49	61.20
4	0.32	56.46	0.04	51.70	53.25	54.84	56.46	58.11	59.80	61.51
4.1	0.31	56.73	0.04	51.93	53.50	55.10	56.73	58.40	60.10	61.83
4.2	0.31	57.01	0.04	52.17	53.75	55.36	57.01	58.69	60.40	62.15
4.3	0.30	57.28	0.04	52.40	54.00	55.62	57.28	58.98	60.71	62.47
4.4	0.29	57.56	0.04	52.64	54.25	55.88	57.56	59.27	61.01	62.79
4.5	0.29	57.83	0.04	52.88	54.49	56.15	57.83	59.56	61.31	63.11
4.6	0.28	58.11	0.04	53.11	54.74	56.41	58.11	59.84	61.62	63.43
4.7	0.28	58.38	0.04	53.35	54.99	56.67	58.38	60.13	61.92	63.75
4.8	0.27	58.66	0.04	53.59	55.24	56.93	58.66	60.42	62.22	64.07
4.9	0.27	58.93	0.04	53.82	55.49	57.19	58.93	60.71	62.53	64.39
5	0.26	59.20	0.04	54.06	55.73	57.45	59.20	61.00	62.83	64.70
5.1	0.26	59.48	0.05	54.29	55.98	57.71	59.48	61.29	63.13	65.02
5.2	0.26	59.75	0.05	54.53	56.23	57.97	59.75	61.57	63.44	65.34
5.3	0.25	60.02	0.05	54.76	56.48	58.23	60.02	61.86	63.74	65.66
5.4	0.25	60.30	0.05	54.99	56.72	58.49	60.30	62.15	64.04	65.97
5.5	0.25	60.57	0.05	55.23	56.97	58.75	60.57	62.43	64.34	66.29
5.6	0.24	60.84	0.05	55.46	57.21	59.01	60.84	62.72	64.64	66.61
5.7	0.24	61.11	0.05	55.70	57.46	59.27	61.11	63.01	64.94	66.92
5.8	0.24	61.39	0.05	55.93	57.70	59.52	61.39	63.29	65.24	67.24
5.9	0.24	61.66	0.05	56.16	57.95	59.78	61.66	63.58	65.54	67.56
6	0.24	61.93	0.05	56.39	58.20	60.04	61.93	63.86	65.84	67.87
6.1	0.23	62.20	0.05	56.63	58.44	60.30	62.20	64.15	66.14	68.19
6.2	0.23	62.47	0.05	56.86	58.68	60.56	62.47	64.43	66.44	68.50
6.3	0.23	62.74	0.05	57.09	58.93	60.81	62.74	64.72	66.74	68.82
6.4	0.23	63.01	0.05	57.32	59.17	61.07	63.01	65.00	67.04	69.13
6.5	0.23	63.28	0.05	57.55	59.41	61.32	63.28	65.29	67.34	69.44
6.6	0.23	63.55	0.05	57.78	59.66	61.58	63.55	65.57	67.64	69.75
6.7	0.23	63.82	0.05	58.01	59.90	61.83	63.82	65.85	67.93	70.07
6.8	0.23	64.09	0.05	58.24	60.14	62.09	64.09	66.13	68.23	70.38
6.9	0.23	64.35	0.05	58.46	60.38	62.34	64.35	66.41	68.52	70.69
7	0.23	64.62	0.05	58.69	60.62	62.59	64.62	66.69	68.82	70.99
7.1	0.23	64.88	0.05	58.92	60.86	62.85	64.88	66.97	69.11	71.30
7.2	0.24	65.15	0.05	59.14	61.09	63.10	65.15	67.25	69.40	71.61
7.3	0.24	65.41	0.05	59.37	61.33	63.35	65.41	67.53	69.69	71.91
7.4	0.24	65.67	0.05	59.59	61.57	63.60	65.67	67.80	69.98	72.22
7.5	0.24	65.93	0.05	59.81	61.80	63.84	65.93	68.08	70.27	72.52
7.6	0.25	66.20	0.05	60.03	62.04	64.09	66.20	68.35	70.56	72.82
7.7	0.25	66.46	0.05	60.25	62.27	64.34	66.46	68.63	70.85	73.12
7.8	0.26	66.71	0.05	60.47	62.50	64.58	66.71	68.90	71.13	73.42
7.9	0.26	66.97	0.05	60.69	62.73	64.83	66.97	69.17	71.42	73.72
8	0.27	67.23	0.05	60.91	62.96	65.07	67.23	69.44	71.70	74.02
8.1	0.28	67.49	0.05	61.12	63.19	65.32	67.49	69.71	71.99	74.31
8.2	0.28	67.74	0.05	61.34	63.42	65.56	67.74	69.98	72.27	74.61
8.3	0.29	68.00	0.05	61.55	63.65	65.80	68.00	70.25	72.55	74.90
8.4	0.30	68.25	0.05	61.77	63.88	66.04	68.25	70.51	72.83	75.20
8.5	0.31	68.51	0.05	61.98	64.11	66.28	68.51	70.78	73.11	75.49
8.6	0.32	68.76	0.05	62.20	64.33	66.52	68.76	71.05	73.39	75.78
8.7	0.33	69.01	0.05	62.41	64.56	66.76	69.01	71.31	73.67	76.07
8.8	0.34	69.27	0.05	62.62	64.79	67.00	69.27	71.58	73.95	76.36
8.9	0.35	69.52	0.05	62.83	65.01	67.24	69.52	71.85	74.22	76.65
9	0.36	69.77	0.05	63.04	65.24	67.48	69.77	72.11	74.50	76.94
9.1	0.37	70.02	0.05	63.25	65.46	67.72	70.02	72.38	74.78	77.23
9.2	0.38	70.27	0.05	63.46	65.69	67.96	70.27	72.64	75.06	77.52
9.3	0.40	70.53	0.05	63.67	65.91	68.20	70.53	72.91	75.33	77.81
9.4	0.41	70.78	0.05	63.88	66.14	68.43	70.78	73.17	75.61	78.10
9.5	0.42	71.03	0.05	64.09	66.36	68.67	71.03	73.44	75.89	78.39
9.6	0.44	71.28	0.05	64.30	66.59	68.91	71.28	73.70	76.17	78.67
9.7	0.45	71.54	0.05	64.51	66.81	69.15	71.54	73.97	76.44	78.96
9.8	0.46	71.79	0.05	64.72	67.04	69.39	71.79	74.23	76.72	79.25
9.9	0.48	72.05	0.05	64.94	67.26	69.63	72.05	74.50	77.00	79.54
10	0.49	72.30	0.05	65.15	67.49	69.87	72.30	74.77	77.28	79.83
10.1	0.51	72.56	0.05	65.36	67.72	70.12	72.56	75.04	77.56	80.12
10.2	0.52	72.81	0.05	65.57	67.95	70.36	72.81	75.31	77.84	80.41
10.3	0.54	73.07	0.05	65.79	68.17	70.60	73.07	75.58	78.12	80.71
10.4	0.55	73.33	0.05	66.00	68.41	70.85	73.33	75.85	78.40	81.00
10.5	0.56	73.59	0.05	66.22	68.64	71.09	73.59	76.12	78.69	81.29
10.6	0.58	73.85	0.05	66.44	68.87	71.34	73.85	76.39	78.97	81.59
10.7	0.59	74.11	0.05	66.65	69.10	71.59	74.11	76.67	79.26	81.89
10.8	0.61	74.38	0.05	66.87	69.34	71.84	74.38	76.95	79.55	82.19
10.9	0.62	74.64	0.05	67.10	69.58	72.09	74.64	77.22	79.84	82.49
11	0.64	74.91	0.05	67.32	69.82	72.35	74.91	77.50	80.13	82.79
11.1	0.65	75.18	0.05	67.54	70.06	72.60	75.18	77.78	80.42	83.09
11.2	0.66	75.45	0.05	67.77	70.30	72.86	75.45	78.07	80.72	83.40
11.3	0.68	75.72	0.05	68.00	70.54	73.12	75.72	78.35	81.01	83.70
11.4	0.69	75.99	0.05	68.23	70.79	73.38	75.99	78.64	81.31	84.01
11.5	0.70	76.27	0.05	68.46	71.04	73.64	76.27	78.93	81.61	84.32
11.6	0.71	76.55	0.05	68.70	71.29	73.90	76.55	79.22	81.91	84.63
11.7	0.72	76.82	0.05	68.93	71.54	74.17	76.82	79.51	82.21	84.95
11.8	0.73	77.10	0.05	69.17	71.79	74.43	77.10	79.80	82.52	85.26
11.9	0.75	77.39	0.05	69.41	72.04	74.70	77.39	80.09	82.82	85.58
12	0.76	77.67	0.05	69.65	72.30	74.97	77.67	80.39	83.13	85.89
12.1	0.77	77.95	0.05	69.89	72.56	75.24	77.95	80.68	83.44	86.21
12.2	0.78	78.24	0.05	70.14	72.81	75.51	78.24	80.98	83.74	86.53
12.3	0.78	78.52	0.05	70.38	73.07	75.79	78.52	81.28	84.05	86.85
12.4	0.79	78.81	0.05	70.63	73.33	76.06	78.81	81.58	84.36	87.17
12.5	0.80	79.09	0.05	70.87	73.59	76.33	79.09	81.87	84.67	87.49
12.6	0.81	79.38	0.05	71.12	73.86	76.61	79.38	82.17	84.98	87.81
12.7	0.82	79.67	0.05	71.37	74.12	76.88	79.67	82.47	85.29	88.13
12.8	0.83	79.96	0.05	71.62	74.38	77.16	79.96	82.77	85.60	88.45
12.9	0.83	80.24	0.05	71.87	74.64	77.44	80.24	83.07	85.91	88.77
13	0.84	80.53	0.05	72.12	74.91	77.71	80.53	83.37	86.22	89.09
13.1	0.85	80.82	0.05	72.37	75.17	77.99	80.82	83.67	86.53	89.41
13.2	0.85	81.11	0.05	72.62	75.43	78.26	81.11	83.96	86.84	89.72
13.3	0.86	81.39	0.05	72.87	75.70	78.54	81.39	84.26	87.14	90.04
13.4	0.87	81.68	0.05	73.12	75.96	78.81	81.68	84.56	87.45	90.35
13.5	0.87	81.96	0.05	73.37	76.22	79.08	81.96	84.85	87.75	90.67
13.6	0.88	82.24	0.05	73.62	76.48	79.36	82.24	85.14	88.06	90.98
13.7	0.88	82.53	0.05	73.87	76.74	79.63	82.53	85.44	88.36	91.29
13.8	0.89	82.81	0.05	74.11	77.00	79.90	82.81	85.73	88.66	91.60
13.9	0.89	83.09	0.05	74.36	77.26	80.17	83.09	86.02	88.96	91.91
14	0.90	83.36	0.05	74.61	77.52	80.43	83.36	86.30	89.25	92.21
14.1	0.90	83.64	0.05	74.85	77.77	80.70	83.64	86.59	89.55	92.51
14.2	0.91	83.91	0.05	75.10	78.03	80.97	83.91	86.87	89.84	92.82
14.3	0.91	84.19	0.05	75.34	78.28	81.23	84.19	87.15	90.13	93.11
14.4	0.92	84.46	0.05	75.59	78.53	81.49	84.46	87.43	90.42	93.41
14.5	0.92	84.73	0.05	75.83	78.79	81.75	84.73	87.71	90.70	93.70
14.6	0.93	85.00	0.05	76.07	79.03	82.01	85.00	87.99	90.99	94.00
14.7	0.93	85.26	0.05	76.30	79.28	82.27	85.26	88.26	91.27	94.28
14.8	0.93	85.53	0.05	76.54	79.53	82.52	85.53	88.53	91.55	94.57
14.9	0.94	85.79	0.05	76.78	79.77	82.78	85.79	88.80	91.83	94.86
15	0.94	86.05	0.05	77.01	80.02	83.03	86.05	89.07	92.10	95.14
15.1	0.94	86.30	0.05	77.25	80.26	83.28	86.30	89.34	92.37	95.42
15.2	0.95	86.56	0.05	77.48	80.50	83.53	86.56	89.60	92.64	95.69
15.3	0.95	86.81	0.05	77.71	80.74	83.77	86.81	89.86	92.91	95.97
15.4	0.96	87.06	0.05	77.93	80.97	84.02	87.06	90.12	93.17	96.24
15.5	0.96	87.31	0.05	78.16	81.21	84.26	87.31	90.37	93.44	96.50
15.6	0.96	87.56	0.05	78.39	81.44	84.50	87.56	90.63	93.70	96.77
15.7	0.97	87.81	0.05	78.61	81.67	84.74	87.81	90.88	93.96	97.04
15.8	0.97	88.05	0.05	78.83	81.90	84.97	88.05	91.13	94.21	97.30
15.9	0.97	88.29	0.05	79.05	82.13	85.21	88.29	91.38	94.47	97.56
16	0.97	88.53	0.05	79.27	82.36	85.44	88.53	91.62	94.72	97.81
16.1	0.98	88.77	0.05	79.49	82.58	85.68	88.77	91.87	94.97	98.07
16.2	0.98	89.01	0.05	79.71	82.81	85.91	89.01	92.11	95.22	98.32
16.3	0.98	89.24	0.05	79.93	83.03	86.14	89.24	92.35	95.46	98.58
16.4	0.98	89.48	0.05	80.14	83.25	86.36	89.48	92.59	95.71	98.83
16.5	0.99	89.71	0.05	80.36	83.47	86.59	89.71	92.83	95.95	99.08
16.6	0.99	89.94	0.05	80.57	83.69	86.82	89.94	93.07	96.20	99.32
16.7	0.99	90.17	0.05	80.78	83.91	87.04	90.17	93.30	96.44	99.57
16.8	0.99	90.40	0.05	80.99	84.13	87.27	90.40	93.54	96.68	99.81
16.9	1.00	90.63	0.05	81.20	84.35	87.49	90.63	93.77	96.92	100.06
17	1.00	90.86	0.05	81.42	84.56	87.71	90.86	94.01	97.15	100.30
17.1	1.00	91.09	0.05	81.62	84.78	87.93	91.09	94.24	97.39	100.54
17.2	1.00	91.31	0.05	81.83	84.99	88.15	91.31	94.47	97.63	100.79
17.3	1.01	91.54	0.05	82.04	85.21	88.37	91.54	94.70	97.86	101.03
17.4	1.01	91.76	0.05	82.25	85.42	88.59	91.76	94.93	98.10	101.27
17.5	1.01	91.99	0.05	82.46	85.64	88.81	91.99	95.16	98.34	101.51
17.6	1.01	92.21	0.05	82.67	85.85	89.03	92.21	95.39	98.57	101.75
17.7	1.02	92.44	0.05	82.88	86.07	89.25	92.44	95.62	98.81	101.99
17.8	1.02	92.66	0.05	83.08	86.28	89.47	92.66	95.85	99.04	102.23
17.9	1.02	92.89	0.05	83.29	86.49	89.69	92.89	96.08	99.28	102.47
18	1.02	92.93	0.05	83.33	86.54	89.74	92.93	96.13	99.32	102.51

**Table 4:** Sitting Height in Non-Hispanic White or Mexican American Females.

	LMS Curves			Percentiles						
Age (years)	L	M	S	3rd	10th	25th	50th	75th	90th	97^th^
2	0.07	50.20	0.04	46.04	47.39	48.77	50.20	51.66	53.15	54.69
2.1	0.10	50.48	0.04	46.28	47.65	49.04	50.48	51.95	53.46	55.01
2.2	0.14	50.76	0.04	46.53	47.90	49.32	50.76	52.25	53.77	55.33
2.3	0.17	51.05	0.04	46.77	48.16	49.59	51.05	52.54	54.08	55.65
2.4	0.20	51.33	0.04	47.01	48.42	49.86	51.33	52.84	54.38	55.96
2.5	0.23	51.61	0.04	47.25	48.67	50.13	51.61	53.13	54.69	56.28
2.6	0.26	51.90	0.04	47.49	48.93	50.40	51.90	53.43	55.00	56.60
2.7	0.29	52.18	0.04	47.73	49.18	50.67	52.18	53.73	55.30	56.91
2.8	0.32	52.46	0.04	47.97	49.44	50.94	52.46	54.02	55.61	57.23
2.9	0.35	52.75	0.04	48.21	49.69	51.21	52.75	54.32	55.92	57.55
3	0.38	53.03	0.04	48.45	49.95	51.47	53.03	54.61	56.22	57.86
3.1	0.41	53.31	0.04	48.69	50.20	51.74	53.31	54.91	56.53	58.18
3.2	0.44	53.59	0.04	48.93	50.46	52.01	53.59	55.20	56.83	58.50
3.3	0.47	53.87	0.04	49.17	50.71	52.28	53.87	55.49	57.14	58.81
3.4	0.50	54.15	0.04	49.40	50.96	52.55	54.15	55.79	57.44	59.12
3.5	0.53	54.43	0.05	49.64	51.21	52.81	54.43	56.08	57.75	59.44
3.6	0.56	54.71	0.05	49.87	51.46	53.08	54.71	56.37	58.05	59.75
3.7	0.59	54.99	0.05	50.11	51.71	53.34	54.99	56.66	58.35	60.06
3.8	0.62	55.27	0.05	50.34	51.96	53.61	55.27	56.95	58.66	60.38
3.9	0.65	55.55	0.05	50.57	52.21	53.87	55.55	57.24	58.96	60.69
4	0.68	55.83	0.05	50.80	52.46	54.14	55.83	57.53	59.26	61.00
4.1	0.71	56.10	0.05	51.03	52.71	54.40	56.10	57.82	59.56	61.31
4.2	0.74	56.38	0.05	51.26	52.95	54.66	56.38	58.11	59.86	61.62
4.3	0.77	56.65	0.05	51.49	53.20	54.92	56.65	58.40	60.16	61.93
4.4	0.80	56.93	0.05	51.72	53.45	55.18	56.93	58.69	60.46	62.23
4.5	0.82	57.20	0.05	51.95	53.69	55.44	57.20	58.97	60.75	62.54
4.6	0.85	57.48	0.05	52.18	53.94	55.70	57.48	59.26	61.05	62.85
4.7	0.88	57.75	0.05	52.41	54.18	55.96	57.75	59.55	61.35	63.16
4.8	0.91	58.03	0.05	52.63	54.42	56.22	58.03	59.83	61.65	63.47
4.9	0.93	58.30	0.05	52.86	54.67	56.48	58.30	60.12	61.94	63.77
5	0.96	58.57	0.05	53.09	54.91	56.74	58.57	60.41	62.24	64.08
5.1	0.98	58.85	0.05	53.31	55.16	57.00	58.85	60.69	62.54	64.39
5.2	1.01	59.12	0.05	53.54	55.40	57.26	59.12	60.98	62.84	64.70
5.3	1.03	59.39	0.05	53.77	55.65	57.52	59.39	61.27	63.14	65.00
5.4	1.05	59.67	0.05	54.00	55.89	57.78	59.67	61.55	63.43	65.31
5.5	1.08	59.94	0.05	54.22	56.13	58.04	59.94	61.84	63.73	65.62
5.6	1.10	60.22	0.05	54.45	56.38	58.30	60.22	62.13	64.03	65.93
5.7	1.12	60.49	0.05	54.68	56.62	58.56	60.49	62.41	64.33	66.24
5.8	1.14	60.77	0.05	54.91	56.87	58.82	60.77	62.70	64.63	66.55
5.9	1.16	61.04	0.05	55.14	57.11	59.08	61.04	62.99	64.93	66.86
6	1.18	61.31	0.05	55.36	57.36	59.34	61.31	63.28	65.23	67.17
6.1	1.19	61.59	0.05	55.59	57.61	59.60	61.59	63.56	65.52	67.47
6.2	1.21	61.87	0.05	55.82	57.85	59.87	61.87	63.85	65.82	67.78
6.3	1.23	62.14	0.05	56.05	58.10	60.13	62.14	64.14	66.12	68.10
6.4	1.24	62.42	0.05	56.28	58.35	60.39	62.42	64.43	66.42	68.41
6.5	1.26	62.69	0.05	56.52	58.59	60.65	62.69	64.72	66.73	68.72
6.6	1.27	62.97	0.05	56.75	58.84	60.92	62.97	65.01	67.03	69.03
6.7	1.28	63.25	0.05	56.98	59.09	61.18	63.25	65.30	67.33	69.34
6.8	1.29	63.53	0.05	57.22	59.34	61.45	63.53	65.59	67.63	69.66
6.9	1.31	63.81	0.05	57.45	59.59	61.71	63.81	65.88	67.94	69.97
7	1.31	64.09	0.05	57.69	59.85	61.98	64.09	66.18	68.24	70.29
7.1	1.32	64.37	0.05	57.93	60.10	62.25	64.37	66.47	68.55	70.61
7.2	1.33	64.65	0.05	58.17	60.35	62.51	64.65	66.76	68.85	70.92
7.3	1.34	64.93	0.05	58.41	60.61	62.78	64.93	67.06	69.16	71.24
7.4	1.34	65.22	0.05	58.65	60.87	63.05	65.22	67.36	69.47	71.56
7.5	1.35	65.50	0.05	58.89	61.12	63.33	65.50	67.65	69.78	71.88
7.6	1.36	65.79	0.05	59.14	61.38	63.60	65.79	67.95	70.09	72.21
7.7	1.36	66.07	0.05	59.38	61.64	63.87	66.07	68.25	70.40	72.53
7.8	1.36	66.36	0.05	59.63	61.90	64.15	66.36	68.55	70.71	72.85
7.9	1.37	66.65	0.05	59.88	62.16	64.42	66.65	68.85	71.02	73.18
8	1.37	66.94	0.05	60.13	62.43	64.70	66.94	69.15	71.34	73.50
8.1	1.37	67.23	0.05	60.38	62.69	64.97	67.23	69.45	71.65	73.83
8.2	1.38	67.52	0.05	60.63	62.95	65.25	67.52	69.75	71.97	74.15
8.3	1.38	67.81	0.05	60.88	63.22	65.53	67.81	70.06	72.28	74.48
8.4	1.38	68.10	0.05	61.13	63.49	65.81	68.10	70.36	72.60	74.80
8.5	1.38	68.39	0.05	61.39	63.76	66.09	68.39	70.67	72.91	75.13
8.6	1.38	68.69	0.05	61.64	64.02	66.37	68.69	70.97	73.23	75.46
8.7	1.38	68.98	0.05	61.90	64.29	66.65	68.98	71.28	73.55	75.79
8.8	1.38	69.28	0.05	62.16	64.57	66.94	69.28	71.58	73.86	76.12
8.9	1.39	69.57	0.05	62.42	64.84	67.22	69.57	71.89	74.18	76.45
9	1.39	69.87	0.05	62.68	65.11	67.50	69.87	72.20	74.50	76.78
9.1	1.39	70.16	0.05	62.94	65.38	67.79	70.16	72.51	74.82	77.11
9.2	1.39	70.46	0.05	63.20	65.66	68.08	70.46	72.82	75.14	77.44
9.3	1.39	70.76	0.05	63.47	65.93	68.36	70.76	73.13	75.46	77.77
9.4	1.39	71.06	0.05	63.73	66.21	68.65	71.06	73.44	75.78	78.10
9.5	1.39	71.36	0.05	64.00	66.49	68.94	71.36	73.75	76.10	78.43
9.6	1.40	71.66	0.05	64.27	66.77	69.23	71.66	74.06	76.42	78.76
9.7	1.40	71.96	0.05	64.53	67.04	69.52	71.96	74.37	76.74	79.09
9.8	1.40	72.26	0.05	64.80	67.32	69.81	72.26	74.68	77.06	79.42
9.9	1.40	72.56	0.05	65.07	67.60	70.10	72.56	74.99	77.38	79.75
10	1.40	72.86	0.05	65.34	67.88	70.39	72.86	75.30	77.70	80.08
10.1	1.41	73.16	0.05	65.61	68.16	70.68	73.16	75.61	78.02	80.41
10.2	1.41	73.46	0.05	65.88	68.44	70.97	73.46	75.92	78.34	80.74
10.3	1.41	73.76	0.05	66.15	68.73	71.26	73.76	76.23	78.66	81.06
10.4	1.41	74.06	0.05	66.42	69.01	71.55	74.06	76.53	78.98	81.39
10.5	1.41	74.36	0.05	66.69	69.29	71.84	74.36	76.84	79.29	81.71
10.6	1.42	74.66	0.05	66.96	69.57	72.13	74.66	77.15	79.61	82.03
10.7	1.42	74.95	0.05	67.23	69.84	72.42	74.95	77.45	79.92	82.35
10.8	1.42	75.25	0.05	67.50	70.12	72.70	75.25	77.75	80.23	82.67
10.9	1.42	75.54	0.05	67.77	70.40	72.99	75.54	78.05	80.54	82.98
11	1.42	75.83	0.05	68.04	70.68	73.27	75.83	78.35	80.84	83.30
11.1	1.42	76.12	0.05	68.30	70.95	73.55	76.12	78.65	81.14	83.61
11.2	1.43	76.41	0.05	68.57	71.22	73.83	76.41	78.94	81.44	83.91
11.3	1.43	76.69	0.05	68.83	71.49	74.11	76.69	79.23	81.74	84.22
11.4	1.43	76.97	0.05	69.10	71.76	74.39	76.97	79.52	82.03	84.51
11.5	1.43	77.25	0.05	69.36	72.03	74.66	77.25	79.80	82.32	84.81
11.6	1.43	77.52	0.05	69.61	72.29	74.93	77.52	80.08	82.61	85.10
11.7	1.43	77.80	0.05	69.87	72.55	75.19	77.80	80.36	82.89	85.39
11.8	1.44	78.06	0.05	70.12	72.81	75.46	78.06	80.63	83.17	85.67
11.9	1.44	78.33	0.05	70.37	73.07	75.72	78.33	80.90	83.44	85.94
12	1.44	78.59	0.05	70.62	73.32	75.97	78.59	81.17	83.71	86.22
12.1	1.44	78.85	0.05	70.87	73.57	76.23	78.85	81.43	83.97	86.48
12.2	1.44	79.10	0.05	71.11	73.82	76.48	79.10	81.68	84.23	86.74
12.3	1.44	79.35	0.05	71.35	74.06	76.72	79.35	81.93	84.48	87.00
12.4	1.44	79.59	0.05	71.59	74.30	76.96	79.59	82.18	84.73	87.25
12.5	1.44	79.83	0.05	71.82	74.53	77.20	79.83	82.42	84.97	87.49
12.6	1.44	80.06	0.05	72.05	74.76	77.43	80.06	82.65	85.21	87.73
12.7	1.45	80.29	0.05	72.28	74.99	77.66	80.29	82.89	85.44	87.97
12.8	1.45	80.52	0.05	72.50	75.21	77.89	80.52	83.11	85.67	88.19
12.9	1.45	80.74	0.05	72.72	75.43	78.11	80.74	83.33	85.89	88.41
13	1.45	80.95	0.05	72.93	75.65	78.32	80.95	83.54	86.10	88.63
13.1	1.45	81.16	0.05	73.15	75.86	78.53	81.16	83.75	86.31	88.84
13.2	1.45	81.37	0.05	73.35	76.07	78.74	81.37	83.96	86.51	89.04
13.3	1.45	81.57	0.05	73.56	76.27	78.94	81.57	84.16	86.71	89.23
13.4	1.45	81.76	0.05	73.76	76.47	79.13	81.76	84.35	86.90	89.42
13.5	1.45	81.95	0.05	73.96	76.66	79.33	81.95	84.54	87.09	89.61
13.6	1.45	82.13	0.05	74.15	76.85	79.51	82.13	84.72	87.27	89.78
13.7	1.45	82.31	0.05	74.34	77.04	79.70	82.31	84.90	87.44	89.96
13.8	1.45	82.49	0.05	74.52	77.22	79.87	82.49	85.07	87.61	90.12
13.9	1.45	82.66	0.05	74.70	77.40	80.05	82.66	85.23	87.77	90.28
14	1.45	82.83	0.05	74.88	77.57	80.22	82.83	85.40	87.93	90.44
14.1	1.46	82.99	0.05	75.06	77.74	80.38	82.99	85.55	88.08	90.58
14.2	1.46	83.14	0.05	75.23	77.91	80.54	83.14	85.70	88.23	90.73
14.3	1.46	83.29	0.05	75.39	78.07	80.70	83.29	85.85	88.37	90.86
14.4	1.46	83.44	0.05	75.56	78.23	80.85	83.44	85.99	88.51	91.00
14.5	1.46	83.58	0.05	75.72	78.38	81.00	83.58	86.13	88.64	91.12
14.6	1.46	83.72	0.05	75.87	78.53	81.15	83.72	86.26	88.77	91.25
14.7	1.46	83.86	0.05	76.03	78.68	81.29	83.86	86.39	88.89	91.36
14.8	1.46	83.99	0.05	76.18	78.82	81.42	83.99	86.52	89.01	91.48
14.9	1.46	84.12	0.05	76.33	78.96	81.56	84.12	86.64	89.13	91.59
15	1.46	84.24	0.05	76.47	79.10	81.69	84.24	86.76	89.24	91.69
15.1	1.46	84.36	0.04	76.61	79.23	81.82	84.36	86.87	89.35	91.79
15.2	1.46	84.48	0.04	76.75	79.37	81.94	84.48	86.98	89.45	91.89
15.3	1.46	84.59	0.04	76.89	79.50	82.06	84.59	87.09	89.55	91.98
15.4	1.46	84.70	0.04	77.03	79.62	82.18	84.70	87.19	89.65	92.07
15.5	1.46	84.81	0.04	77.16	79.75	82.30	84.81	87.29	89.74	92.16
15.6	1.46	84.92	0.04	77.29	79.87	82.41	84.92	87.39	89.83	92.24
15.7	1.46	85.02	0.04	77.42	79.99	82.52	85.02	87.48	89.92	92.32
15.8	1.46	85.12	0.04	77.54	80.11	82.63	85.12	87.58	90.00	92.40
15.9	1.46	85.22	0.04	77.67	80.22	82.74	85.22	87.67	90.09	92.47
16	1.46	85.32	0.04	77.79	80.34	82.84	85.32	87.76	90.17	92.55
16.1	1.46	85.41	0.04	77.91	80.45	82.95	85.41	87.84	90.24	92.62
16.2	1.46	85.50	0.04	78.03	80.56	83.05	85.50	87.93	90.32	92.69
16.3	1.46	85.60	0.04	78.15	80.67	83.15	85.60	88.01	90.40	92.75
16.4	1.46	85.69	0.04	78.27	80.78	83.25	85.69	88.09	90.47	92.82
16.5	1.46	85.78	0.04	78.39	80.89	83.35	85.78	88.17	90.54	92.88
16.6	1.46	85.87	0.04	78.51	80.99	83.45	85.87	88.25	90.61	92.95
16.7	1.46	85.95	0.04	78.62	81.10	83.54	85.95	88.33	90.69	93.01
16.8	1.46	86.04	0.04	78.74	81.21	83.64	86.04	88.41	90.76	93.07
16.9	1.45	86.13	0.04	78.85	81.31	83.73	86.13	88.49	90.82	93.13
17	1.45	86.21	0.04	78.97	81.42	83.83	86.21	88.57	90.89	93.19
17.1	1.45	86.30	0.04	79.08	81.52	83.93	86.30	88.64	90.96	93.25
17.2	1.45	86.38	0.04	79.20	81.62	84.02	86.38	88.72	91.03	93.31
17.3	1.45	86.47	0.04	79.31	81.73	84.11	86.47	88.80	91.10	93.37
17.4	1.45	86.56	0.04	79.43	81.83	84.21	86.56	88.87	91.16	93.43
17.5	1.45	86.64	0.04	79.54	81.94	84.30	86.64	88.95	91.23	93.49
17.6	1.45	86.73	0.04	79.66	82.04	84.40	86.73	89.03	91.30	93.55
17.7	1.45	86.81	0.04	79.77	82.15	84.49	86.81	89.10	91.37	93.61
17.8	1.45	86.90	0.04	79.88	82.25	84.59	86.90	89.18	91.43	93.67
17.9	1.45	86.98	0.04	80.00	82.36	84.68	86.98	89.25	91.50	93.72
18	1.44	87.00	0.04	80.02	82.38	84.70	87.00	89.27	91.51	93.74

**Table 5:** Sitting Height in Non-Hispanic Black Males.

	LMS Curves			Percentiles						
Age (years)	L	M	S	3rd	10th	25th	50th	75th	90th	97^th^
2	−0.29	50.05	0.04	46.16	47.41	48.71	50.05	51.45	52.89	54.39
2.1	−0.28	50.36	0.04	46.42	47.69	49.00	50.36	51.76	53.22	54.73
2.2	−0.27	50.66	0.04	46.68	47.96	49.29	50.66	52.08	53.55	55.08
2.3	−0.26	50.96	0.04	46.94	48.24	49.58	50.96	52.40	53.88	55.42
2.4	−0.24	51.26	0.04	47.20	48.51	49.86	51.26	52.71	54.21	55.77
2.5	−0.23	51.57	0.04	47.47	48.79	50.15	51.57	53.03	54.54	56.11
2.6	−0.22	51.87	0.04	47.73	49.06	50.44	51.87	53.34	54.87	56.46
2.7	−0.21	52.17	0.04	47.99	49.33	50.73	52.17	53.66	55.20	56.80
2.8	−0.20	52.47	0.04	48.25	49.61	51.02	52.47	53.98	55.53	57.15
2.9	−0.19	52.77	0.04	48.51	49.88	51.30	52.77	54.29	55.86	57.49
3	−0.18	53.07	0.04	48.77	50.15	51.59	53.07	54.61	56.19	57.84
3.1	−0.17	53.37	0.04	49.03	50.43	51.88	53.37	54.92	56.52	58.18
3.2	−0.16	53.67	0.04	49.28	50.70	52.16	53.67	55.24	56.85	58.52
3.3	−0.14	53.97	0.04	49.54	50.97	52.45	53.97	55.55	57.18	58.87
3.4	−0.13	54.27	0.04	49.80	51.24	52.73	54.27	55.87	57.51	59.21
3.5	−0.12	54.57	0.04	50.05	51.51	53.02	54.57	56.18	57.84	59.56
3.6	−0.11	54.87	0.04	50.31	51.78	53.30	54.87	56.49	58.17	59.90
3.7	−0.10	55.17	0.04	50.56	52.05	53.59	55.17	56.81	58.50	60.24
3.8	−0.09	55.47	0.04	50.82	52.32	53.87	55.47	57.12	58.82	60.58
3.9	−0.07	55.76	0.04	51.07	52.58	54.15	55.76	57.43	59.15	60.92
4	−0.06	56.06	0.04	51.32	52.85	54.43	56.06	57.74	59.47	61.26
4.1	−0.05	56.35	0.04	51.57	53.11	54.71	56.35	58.04	59.79	61.60
4.2	−0.04	56.64	0.04	51.82	53.38	54.98	56.64	58.35	60.11	61.93
4.3	−0.02	56.93	0.04	52.06	53.64	55.26	56.93	58.66	60.43	62.27
4.4	−0.01	57.22	0.04	52.31	53.90	55.53	57.22	58.96	60.75	62.60
4.5	0.00	57.51	0.05	52.55	54.16	55.81	57.51	59.26	61.07	62.93
4.6	0.02	57.80	0.05	52.80	54.41	56.08	57.80	59.56	61.38	63.26
4.7	0.03	58.08	0.05	53.04	54.67	56.35	58.08	59.86	61.70	63.59
4.8	0.05	58.36	0.05	53.27	54.92	56.62	58.36	60.16	62.01	63.91
4.9	0.06	58.65	0.05	53.51	55.17	56.88	58.65	60.46	62.32	64.24
5	0.08	58.93	0.05	53.75	55.42	57.15	58.93	60.75	62.63	64.56
5.1	0.09	59.20	0.05	53.98	55.67	57.41	59.20	61.04	62.94	64.88
5.2	0.11	59.48	0.05	54.21	55.92	57.68	59.48	61.34	63.24	65.20
5.3	0.12	59.76	0.05	54.44	56.17	57.94	59.76	61.62	63.54	65.52
5.4	0.14	60.03	0.05	54.67	56.41	58.19	60.03	61.91	63.85	65.83
5.5	0.16	60.30	0.05	54.90	56.65	58.45	60.30	62.20	64.15	66.14
5.6	0.18	60.57	0.05	55.12	56.89	58.71	60.57	62.48	64.44	66.46
5.7	0.19	60.84	0.05	55.35	57.13	58.96	60.84	62.77	64.74	66.76
5.8	0.21	61.11	0.05	55.57	57.37	59.22	61.11	63.05	65.04	67.07
5.9	0.23	61.37	0.05	55.79	57.61	59.47	61.37	63.33	65.33	67.38
6	0.25	61.64	0.05	56.01	57.84	59.72	61.64	63.61	65.62	67.68
6.1	0.27	61.90	0.05	56.23	58.07	59.97	61.90	63.88	65.91	67.98
6.2	0.29	62.16	0.05	56.44	58.31	60.21	62.16	64.16	66.20	68.28
6.3	0.31	62.43	0.05	56.66	58.54	60.46	62.43	64.43	66.49	68.58
6.4	0.33	62.68	0.05	56.87	58.77	60.71	62.68	64.71	66.77	68.88
6.5	0.35	62.94	0.05	57.09	59.00	60.95	62.94	64.98	67.05	69.17
6.6	0.37	63.20	0.05	57.30	59.23	61.19	63.20	65.25	67.34	69.47
6.7	0.40	63.45	0.05	57.51	59.45	61.43	63.45	65.52	67.62	69.76
6.8	0.42	63.71	0.05	57.72	59.68	61.67	63.71	65.78	67.90	70.05
6.9	0.44	63.96	0.05	57.92	59.90	61.91	63.96	66.05	68.17	70.33
7	0.46	64.21	0.05	58.13	60.12	62.15	64.21	66.31	68.45	70.62
7.1	0.49	64.46	0.05	58.34	60.34	62.38	64.46	66.57	68.72	70.90
7.2	0.51	64.71	0.05	58.54	60.56	62.62	64.71	66.83	68.99	71.18
7.3	0.53	64.96	0.05	58.74	60.78	62.85	64.96	67.09	69.26	71.46
7.4	0.56	65.20	0.05	58.94	61.00	63.08	65.20	67.35	69.53	71.74
7.5	0.58	65.45	0.05	59.14	61.21	63.31	65.45	67.61	69.80	72.02
7.6	0.61	65.69	0.05	59.34	61.43	63.54	65.69	67.86	70.06	72.29
7.7	0.63	65.93	0.05	59.54	61.64	63.77	65.93	68.12	70.33	72.57
7.8	0.66	66.17	0.05	59.73	61.85	64.00	66.17	68.37	70.59	72.84
7.9	0.68	66.41	0.05	59.93	62.07	64.23	66.41	68.62	70.85	73.11
8	0.71	66.65	0.05	60.12	62.28	64.46	66.65	68.87	71.12	73.38
8.1	0.73	66.89	0.05	60.32	62.49	64.68	66.89	69.13	71.38	73.65
8.2	0.76	67.13	0.05	60.51	62.70	64.91	67.13	69.38	71.64	73.92
8.3	0.79	67.37	0.05	60.70	62.91	65.13	67.37	69.63	71.90	74.18
8.4	0.81	67.61	0.05	60.90	63.12	65.36	67.61	69.88	72.16	74.45
8.5	0.84	67.85	0.05	61.09	63.33	65.58	67.85	70.13	72.42	74.72
8.6	0.86	68.09	0.05	61.28	63.54	65.81	68.09	70.38	72.68	74.98
8.7	0.89	68.33	0.05	61.48	63.75	66.03	68.33	70.63	72.93	75.25
8.8	0.92	68.57	0.05	61.67	63.96	66.26	68.57	70.88	73.19	75.52
8.9	0.94	68.80	0.05	61.86	64.17	66.49	68.80	71.13	73.45	75.78
9	0.97	69.04	0.05	62.06	64.38	66.71	69.04	71.38	73.71	76.05
9.1	0.99	69.28	0.05	62.25	64.60	66.94	69.28	71.63	73.97	76.32
9.2	1.02	69.52	0.05	62.45	64.81	67.17	69.52	71.88	74.23	76.59
9.3	1.04	69.76	0.05	62.64	65.02	67.39	69.76	72.13	74.49	76.85
9.4	1.07	70.01	0.05	62.84	65.23	67.62	70.01	72.38	74.75	77.12
9.5	1.09	70.25	0.05	63.04	65.45	67.85	70.25	72.64	75.02	77.39
9.6	1.12	70.49	0.05	63.24	65.67	68.08	70.49	72.89	75.28	77.66
9.7	1.14	70.74	0.05	63.44	65.88	68.32	70.74	73.15	75.54	77.93
9.8	1.16	70.98	0.05	63.64	66.10	68.55	70.98	73.40	75.81	78.20
9.9	1.19	71.23	0.05	63.84	66.32	68.78	71.23	73.66	76.07	78.48
10	1.21	71.48	0.05	64.04	66.54	69.02	71.48	73.92	76.34	78.75
10.1	1.23	71.72	0.05	64.25	66.76	69.25	71.72	74.18	76.61	79.02
10.2	1.25	71.97	0.05	64.46	66.99	69.49	71.97	74.44	76.88	79.30
10.3	1.27	72.23	0.05	64.66	67.21	69.73	72.23	74.70	77.15	79.58
10.4	1.29	72.48	0.05	64.88	67.44	69.97	72.48	74.96	77.42	79.86
10.5	1.31	72.73	0.05	65.09	67.67	70.21	72.73	75.23	77.69	80.14
10.6	1.33	72.99	0.05	65.30	67.90	70.46	72.99	75.49	77.97	80.42
10.7	1.35	73.25	0.05	65.52	68.13	70.70	73.25	75.76	78.24	80.70
10.8	1.37	73.51	0.05	65.74	68.36	70.95	73.51	76.03	78.52	80.98
10.9	1.39	73.77	0.05	65.96	68.60	71.20	73.77	76.30	78.80	81.27
11	1.40	74.03	0.05	66.18	68.83	71.45	74.03	76.57	79.08	81.56
11.1	1.42	74.29	0.05	66.40	69.07	71.70	74.29	76.84	79.36	81.84
11.2	1.44	74.56	0.05	66.63	69.31	71.96	74.56	77.12	79.64	82.13
11.3	1.46	74.82	0.05	66.85	69.55	72.21	74.82	77.39	79.92	82.42
11.4	1.48	75.09	0.05	67.08	69.80	72.47	75.09	77.67	80.21	82.71
11.5	1.49	75.36	0.05	67.31	70.04	72.72	75.36	77.95	80.49	83.00
11.6	1.51	75.63	0.05	67.54	70.29	72.98	75.63	78.22	80.78	83.29
11.7	1.53	75.90	0.05	67.77	70.54	73.24	75.90	78.50	81.06	83.58
11.8	1.55	76.17	0.05	68.01	70.79	73.50	76.17	78.78	81.35	83.88
11.9	1.56	76.44	0.05	68.25	71.04	73.77	76.44	79.06	81.64	84.17
12	1.58	76.71	0.05	68.48	71.29	74.03	76.71	79.34	81.93	84.46
12.1	1.60	76.99	0.05	68.72	71.54	74.29	76.99	79.63	82.21	84.75
12.2	1.61	77.26	0.05	68.96	71.79	74.56	77.26	79.91	82.50	85.05
12.3	1.63	77.54	0.05	69.21	72.05	74.83	77.54	80.19	82.79	85.34
12.4	1.65	77.81	0.05	69.45	72.31	75.09	77.81	80.48	83.08	85.64
12.5	1.66	78.09	0.05	69.69	72.56	75.36	78.09	80.76	83.37	85.93
12.6	1.68	78.37	0.05	69.94	72.82	75.63	78.37	81.04	83.66	86.22
12.7	1.70	78.64	0.05	70.18	73.08	75.90	78.64	81.33	83.95	86.51
12.8	1.71	78.92	0.05	70.43	73.34	76.17	78.92	81.61	84.24	86.81
12.9	1.73	79.20	0.05	70.68	73.60	76.43	79.20	81.89	84.52	87.10
13	1.74	79.47	0.05	70.92	73.85	76.70	79.47	82.17	84.81	87.39
13.1	1.76	79.75	0.05	71.17	74.11	76.97	79.75	82.45	85.09	87.67
13.2	1.77	80.02	0.05	71.42	74.37	77.24	80.02	82.73	85.38	87.96
13.3	1.79	80.29	0.05	71.67	74.63	77.50	80.29	83.01	85.66	88.24
13.4	1.80	80.56	0.05	71.91	74.88	77.77	80.56	83.28	85.94	88.52
13.5	1.82	80.83	0.05	72.16	75.14	78.03	80.83	83.56	86.21	88.80
13.6	1.83	81.10	0.05	72.40	75.40	78.29	81.10	83.83	86.49	89.08
13.7	1.85	81.37	0.05	72.65	75.65	78.55	81.37	84.10	86.76	89.35
13.8	1.86	81.63	0.05	72.89	75.90	78.81	81.63	84.37	87.03	89.62
13.9	1.88	81.89	0.05	73.14	76.15	79.07	81.89	84.63	87.30	89.89
14	1.89	82.15	0.05	73.38	76.40	79.32	82.15	84.89	87.56	90.16
14.1	1.91	82.41	0.05	73.62	76.65	79.58	82.41	85.15	87.82	90.42
14.2	1.92	82.66	0.05	73.86	76.90	79.83	82.66	85.41	88.08	90.68
14.3	1.93	82.91	0.05	74.10	77.14	80.08	82.91	85.66	88.33	90.93
14.4	1.95	83.16	0.05	74.33	77.38	80.32	83.16	85.92	88.59	91.18
14.5	1.96	83.41	0.05	74.57	77.62	80.57	83.41	86.16	88.83	91.43
14.6	1.97	83.65	0.05	74.80	77.86	80.81	83.65	86.41	89.08	91.67
14.7	1.99	83.89	0.05	75.03	78.10	81.04	83.89	86.65	89.32	91.91
14.8	2.00	84.13	0.05	75.26	78.33	81.28	84.13	86.89	89.56	92.15
14.9	2.01	84.36	0.05	75.49	78.56	81.51	84.36	87.12	89.79	92.38
15	2.03	84.59	0.05	75.71	78.79	81.74	84.59	87.35	90.02	92.61
15.1	2.04	84.82	0.05	75.93	79.01	81.97	84.82	87.58	90.25	92.83
15.2	2.05	85.05	0.05	76.15	79.24	82.20	85.05	87.80	90.47	93.06
15.3	2.07	85.27	0.05	76.37	79.46	82.42	85.27	88.02	90.69	93.27
15.4	2.08	85.49	0.05	76.59	79.68	82.64	85.49	88.24	90.91	93.49
15.5	2.09	85.71	0.05	76.81	79.89	82.86	85.71	88.46	91.12	93.70
15.6	2.11	85.92	0.05	77.02	80.11	83.07	85.92	88.67	91.33	93.90
15.7	2.12	86.13	0.05	77.23	80.32	83.29	86.13	88.88	91.54	94.11
15.8	2.13	86.34	0.05	77.44	80.53	83.50	86.34	89.09	91.74	94.31
15.9	2.15	86.55	0.05	77.65	80.74	83.71	86.55	89.30	91.95	94.51
16	2.16	86.76	0.05	77.86	80.95	83.91	86.76	89.50	92.15	94.71
16.1	2.17	86.96	0.05	78.06	81.16	84.12	86.96	89.70	92.34	94.90
16.2	2.19	87.16	0.05	78.27	81.36	84.32	87.16	89.90	92.54	95.09
16.3	2.20	87.37	0.05	78.47	81.57	84.53	87.37	90.10	92.74	95.29
16.4	2.21	87.57	0.05	78.68	81.77	84.73	87.57	90.30	92.93	95.47
16.5	2.23	87.76	0.05	78.88	81.97	84.93	87.76	90.49	93.12	95.66
16.6	2.24	87.96	0.05	79.08	82.17	85.13	87.96	90.69	93.31	95.85
16.7	2.25	88.16	0.05	79.28	82.38	85.33	88.16	90.88	93.50	96.03
16.8	2.27	88.35	0.05	79.49	82.58	85.53	88.35	91.07	93.69	96.22
16.9	2.28	88.55	0.05	79.69	82.78	85.72	88.55	91.26	93.88	96.40
17	2.29	88.74	0.05	79.89	82.98	85.92	88.74	91.45	94.06	96.58
17.1	2.30	88.94	0.05	80.09	83.17	86.12	88.94	91.64	94.25	96.76
17.2	2.32	89.13	0.05	80.29	83.37	86.32	89.13	91.83	94.43	96.94
17.3	2.33	89.32	0.05	80.49	83.57	86.51	89.32	92.02	94.62	97.13
17.4	2.34	89.52	0.05	80.69	83.77	86.71	89.52	92.21	94.80	97.31
17.5	2.36	89.71	0.05	80.89	83.97	86.90	89.71	92.40	94.99	97.49
17.6	2.37	89.90	0.05	81.09	84.17	87.10	89.90	92.59	95.17	97.67
17.7	2.38	90.10	0.05	81.29	84.37	87.30	90.10	92.78	95.36	97.85
17.8	2.39	90.29	0.05	81.49	84.57	87.49	90.29	92.97	95.54	98.03
17.9	2.41	90.48	0.05	81.69	84.77	87.69	90.48	93.16	95.73	98.21
18	2.41	90.52	0.05	81.73	84.81	87.73	90.52	93.19	95.76	98.24

**Table 6:** Sitting Height in Non-Hispanic Black Females.

	LMS Curves			Percentiles						
Age (years)	L	M	S	3rd	10th	25th	50th	75th	90th	97^th^
2	0.32	48.87	0.04	44.97	46.25	47.55	48.87	50.22	51.59	52.99
2.1	0.31	49.20	0.04	45.26	46.55	47.86	49.20	50.56	51.95	53.37
2.2	0.30	49.52	0.04	45.54	46.84	48.17	49.52	50.90	52.31	53.74
2.3	0.28	49.85	0.04	45.83	47.14	48.48	49.85	51.24	52.66	54.11
2.4	0.27	50.17	0.04	46.11	47.44	48.79	50.17	51.58	53.02	54.49
2.5	0.26	50.50	0.04	46.39	47.73	49.10	50.50	51.92	53.38	54.86
2.6	0.25	50.82	0.04	46.68	48.03	49.41	50.82	52.26	53.73	55.24
2.7	0.24	51.14	0.04	46.96	48.32	49.72	51.14	52.60	54.09	55.61
2.8	0.22	51.47	0.04	47.24	48.62	50.03	51.47	52.94	54.45	55.98
2.9	0.21	51.79	0.04	47.52	48.91	50.34	51.79	53.28	54.80	56.36
3	0.20	52.11	0.04	47.80	49.21	50.64	52.11	53.62	55.16	56.73
3.1	0.19	52.44	0.04	48.08	49.50	50.95	52.44	53.96	55.51	57.10
3.2	0.18	52.76	0.04	48.36	49.79	51.26	52.76	54.30	55.87	57.48
3.3	0.17	53.08	0.04	48.64	50.09	51.56	53.08	54.63	56.22	57.85
3.4	0.16	53.40	0.04	48.92	50.38	51.87	53.40	54.97	56.58	58.22
3.5	0.14	53.72	0.04	49.20	50.67	52.17	53.72	55.30	56.93	58.59
3.6	0.13	54.04	0.04	49.47	50.96	52.48	54.04	55.64	57.28	58.96
3.7	0.12	54.36	0.04	49.75	51.25	52.78	54.36	55.97	57.63	59.33
3.8	0.11	54.67	0.04	50.03	51.53	53.08	54.67	56.31	57.98	59.70
3.9	0.10	54.99	0.04	50.30	51.82	53.38	54.99	56.64	58.33	60.07
4	0.09	55.30	0.04	50.57	52.11	53.68	55.30	56.97	58.68	60.44
4.1	0.08	55.62	0.04	50.84	52.39	53.98	55.62	57.30	59.03	60.80
4.2	0.07	55.93	0.04	51.11	52.67	54.28	55.93	57.63	59.37	61.17
4.3	0.06	56.24	0.05	51.38	52.96	54.57	56.24	57.95	59.72	61.53
4.4	0.05	56.55	0.05	51.65	53.24	54.87	56.55	58.28	60.06	61.89
4.5	0.04	56.86	0.05	51.91	53.51	55.16	56.86	58.60	60.40	62.25
4.6	0.04	57.16	0.05	52.18	53.79	55.45	57.16	58.93	60.74	62.61
4.7	0.03	57.47	0.05	52.44	54.07	55.74	57.47	59.25	61.08	62.97
4.8	0.02	57.77	0.05	52.70	54.34	56.03	57.77	59.57	61.42	63.32
4.9	0.01	58.08	0.05	52.96	54.62	56.32	58.08	59.89	61.75	63.67
5	0.01	58.38	0.05	53.22	54.89	56.61	58.38	60.20	62.09	64.03
5.1	0.00	58.68	0.05	53.48	55.16	56.89	58.68	60.52	62.42	64.38
5.2	0.00	58.98	0.05	53.74	55.43	57.17	58.98	60.83	62.75	64.73
5.3	−0.01	59.27	0.05	53.99	55.70	57.46	59.27	61.15	63.08	65.08
5.4	−0.01	59.57	0.05	54.25	55.97	57.74	59.57	61.46	63.41	65.42
5.5	−0.02	59.86	0.05	54.50	56.23	58.02	59.86	61.77	63.74	65.77
5.6	−0.02	60.16	0.05	54.75	56.50	58.30	60.16	62.08	64.06	66.11
5.7	−0.03	60.45	0.05	55.00	56.76	58.58	60.45	62.39	64.39	66.46
5.8	−0.03	60.75	0.05	55.26	57.03	58.86	60.75	62.70	64.71	66.80
5.9	−0.03	61.04	0.05	55.51	57.29	59.13	61.04	63.00	65.04	67.14
6	−0.03	61.33	0.05	55.75	57.55	59.41	61.33	63.31	65.36	67.48
6.1	−0.03	61.62	0.05	56.00	57.81	59.68	61.62	63.62	65.68	67.82
6.2	−0.03	61.91	0.05	56.25	58.07	59.96	61.91	63.92	66.00	68.15
6.3	−0.03	62.19	0.05	56.50	58.33	60.23	62.19	64.22	66.32	68.49
6.4	−0.03	62.48	0.05	56.74	58.59	60.50	62.48	64.53	66.64	68.82
6.5	−0.03	62.77	0.05	56.99	58.85	60.78	62.77	64.83	66.96	69.16
6.6	−0.03	63.06	0.05	57.23	59.11	61.05	63.06	65.13	67.27	69.49
6.7	−0.02	63.34	0.05	57.48	59.37	61.32	63.34	65.43	67.59	69.82
6.8	−0.02	63.63	0.05	57.72	59.62	61.59	63.63	65.73	67.91	70.15
6.9	−0.01	63.91	0.05	57.96	59.88	61.86	63.91	66.03	68.22	70.48
7	−0.01	64.20	0.05	58.21	60.14	62.13	64.20	66.33	68.53	70.81
7.1	0.00	64.48	0.05	58.45	60.39	62.41	64.48	66.63	68.85	71.14
7.2	0.01	64.77	0.05	58.69	60.65	62.68	64.77	66.93	69.16	71.47
7.3	0.02	65.05	0.05	58.93	60.91	62.95	65.05	67.23	69.47	71.79
7.4	0.03	65.34	0.05	59.17	61.16	63.22	65.34	67.52	69.78	72.12
7.5	0.04	65.62	0.05	59.42	61.42	63.49	65.62	67.82	70.09	72.44
7.6	0.05	65.90	0.05	59.66	61.68	63.76	65.90	68.12	70.40	72.76
7.7	0.06	66.19	0.05	59.90	61.93	64.03	66.19	68.42	70.71	73.08
7.8	0.08	66.47	0.05	60.14	62.19	64.30	66.47	68.71	71.02	73.41
7.9	0.09	66.75	0.05	60.39	62.44	64.57	66.75	69.01	71.33	73.73
8	0.10	67.04	0.05	60.63	62.70	64.84	67.04	69.31	71.64	74.05
8.1	0.12	67.32	0.05	60.87	62.96	65.11	67.32	69.60	71.95	74.37
8.2	0.13	67.61	0.05	61.11	63.22	65.38	67.61	69.90	72.26	74.69
8.3	0.14	67.89	0.05	61.36	63.47	65.65	67.89	70.19	72.57	75.01
8.4	0.16	68.17	0.05	61.60	63.73	65.92	68.17	70.49	72.87	75.32
8.5	0.18	68.46	0.05	61.85	63.99	66.19	68.46	70.79	73.18	75.64
8.6	0.19	68.74	0.05	62.09	64.25	66.46	68.74	71.08	73.49	75.96
8.7	0.21	69.03	0.05	62.34	64.51	66.74	69.03	71.38	73.79	76.27
8.8	0.22	69.31	0.05	62.58	64.76	67.01	69.31	71.67	74.10	76.59
8.9	0.24	69.59	0.05	62.83	65.02	67.28	69.59	71.97	74.41	76.90
9	0.26	69.88	0.05	63.07	65.28	67.55	69.88	72.26	74.71	77.22
9.1	0.28	70.16	0.05	63.32	65.54	67.82	70.16	72.56	75.01	77.53
9.2	0.29	70.45	0.05	63.56	65.80	68.10	70.45	72.85	75.32	77.84
9.3	0.31	70.73	0.05	63.81	66.06	68.37	70.73	73.15	75.62	78.15
9.4	0.33	71.02	0.05	64.06	66.32	68.64	71.02	73.44	75.92	78.46
9.5	0.35	71.30	0.05	64.31	66.59	68.92	71.30	73.74	76.23	78.77
9.6	0.37	71.58	0.05	64.55	66.85	69.19	71.58	74.03	76.53	79.08
9.7	0.39	71.87	0.05	64.80	67.11	69.46	71.87	74.32	76.83	79.39
9.8	0.41	72.15	0.05	65.05	67.37	69.74	72.15	74.62	77.13	79.69
9.9	0.43	72.43	0.05	65.30	67.63	70.01	72.43	74.91	77.43	80.00
10	0.44	72.72	0.05	65.55	67.89	70.28	72.72	75.20	77.73	80.30
10.1	0.46	73.00	0.05	65.80	68.16	70.56	73.00	75.49	78.02	80.60
10.2	0.48	73.28	0.05	66.05	68.42	70.83	73.28	75.78	78.32	80.91
10.3	0.50	73.56	0.05	66.30	68.68	71.10	73.56	76.07	78.62	81.20
10.4	0.52	73.85	0.05	66.55	68.94	71.37	73.85	76.36	78.91	81.50
10.5	0.54	74.13	0.05	66.80	69.20	71.64	74.13	76.65	79.20	81.80
10.6	0.57	74.40	0.05	67.05	69.46	71.91	74.40	76.93	79.49	82.09
10.7	0.59	74.68	0.05	67.29	69.72	72.18	74.68	77.21	79.78	82.38
10.8	0.61	74.96	0.05	67.54	69.98	72.45	74.96	77.50	80.07	82.67
10.9	0.63	75.23	0.05	67.79	70.24	72.72	75.23	77.78	80.35	82.96
11	0.65	75.50	0.05	68.03	70.49	72.98	75.50	78.06	80.64	83.24
11.1	0.67	75.78	0.05	68.28	70.75	73.25	75.78	78.33	80.91	83.52
11.2	0.69	76.04	0.05	68.52	71.00	73.51	76.04	78.60	81.19	83.80
11.3	0.71	76.31	0.05	68.76	71.25	73.77	76.31	78.87	81.46	84.07
11.4	0.74	76.57	0.05	69.00	71.50	74.03	76.57	79.14	81.73	84.34
11.5	0.76	76.83	0.05	69.24	71.75	74.28	76.83	79.40	82.00	84.61
11.6	0.78	77.09	0.05	69.47	71.99	74.53	77.09	79.66	82.26	84.87
11.7	0.81	77.34	0.05	69.70	72.23	74.78	77.34	79.92	82.51	85.12
11.8	0.83	77.59	0.05	69.93	72.47	75.02	77.59	80.17	82.77	85.37
11.9	0.86	77.83	0.05	70.16	72.70	75.26	77.83	80.42	83.01	85.62
12	0.88	78.08	0.05	70.38	72.93	75.50	78.08	80.66	83.26	85.86
12.1	0.91	78.31	0.05	70.60	73.16	75.73	78.31	80.90	83.49	86.09
12.2	0.94	78.54	0.05	70.81	73.38	75.96	78.54	81.13	83.72	86.32
12.3	0.97	78.77	0.05	71.02	73.60	76.19	78.77	81.36	83.95	86.54
12.4	1.00	78.99	0.05	71.23	73.82	76.41	78.99	81.58	84.17	86.76
12.5	1.03	79.21	0.05	71.43	74.03	76.62	79.21	81.80	84.39	86.97
12.6	1.06	79.43	0.05	71.63	74.23	76.83	79.43	82.01	84.60	87.17
12.7	1.10	79.63	0.05	71.82	74.44	77.04	79.63	82.22	84.80	87.37
12.8	1.13	79.84	0.05	72.01	74.63	77.24	79.84	82.42	84.99	87.56
12.9	1.17	80.03	0.05	72.20	74.82	77.44	80.03	82.62	85.18	87.74
13	1.21	80.22	0.05	72.37	75.01	77.63	80.22	82.81	85.37	87.92
13.1	1.25	80.41	0.05	72.55	75.19	77.81	80.41	82.99	85.55	88.09
13.2	1.29	80.59	0.05	72.72	75.37	77.99	80.59	83.17	85.72	88.25
13.3	1.33	80.77	0.05	72.88	75.54	78.17	80.77	83.34	85.89	88.41
13.4	1.37	80.94	0.05	73.04	75.71	78.34	80.94	83.51	86.05	88.56
13.5	1.42	81.11	0.05	73.20	75.87	78.51	81.11	83.67	86.20	88.70
13.6	1.46	81.27	0.05	73.35	76.03	78.67	81.27	83.83	86.35	88.84
13.7	1.51	81.42	0.05	73.50	76.18	78.82	81.42	83.98	86.49	88.97
13.8	1.56	81.57	0.05	73.64	76.33	78.98	81.57	84.12	86.63	89.10
13.9	1.61	81.72	0.05	73.77	76.48	79.12	81.72	84.26	86.76	89.22
14	1.66	81.86	0.05	73.91	76.62	79.27	81.86	84.40	86.89	89.34
14.1	1.71	82.00	0.05	74.03	76.75	79.41	82.00	84.53	87.01	89.45
14.2	1.76	82.13	0.05	74.16	76.89	79.54	82.13	84.66	87.13	89.55
14.3	1.81	82.26	0.05	74.28	77.01	79.67	82.26	84.78	87.24	89.65
14.4	1.87	82.38	0.05	74.40	77.14	79.80	82.38	84.90	87.35	89.75
14.5	1.92	82.50	0.05	74.51	77.26	79.92	82.50	85.01	87.46	89.84
14.6	1.98	82.62	0.05	74.62	77.37	80.04	82.62	85.12	87.55	89.92
14.7	2.04	82.73	0.05	74.72	77.49	80.15	82.73	85.23	87.65	90.00
14.8	2.10	82.84	0.05	74.82	77.60	80.26	82.84	85.33	87.74	90.08
14.9	2.16	82.94	0.05	74.92	77.70	80.37	82.94	85.43	87.83	90.16
15	2.22	83.05	0.05	75.02	77.81	80.48	83.05	85.52	87.91	90.23
15.1	2.28	83.14	0.05	75.11	77.91	80.58	83.14	85.61	87.99	90.29
15.2	2.34	83.24	0.05	75.20	78.00	80.68	83.24	85.70	88.07	90.36
15.3	2.41	83.33	0.05	75.28	78.10	80.77	83.33	85.79	88.14	90.42
15.4	2.47	83.42	0.04	75.37	78.19	80.87	83.42	85.87	88.21	90.47
15.5	2.53	83.51	0.04	75.45	78.28	80.96	83.51	85.95	88.28	90.53
15.6	2.60	83.59	0.04	75.52	78.36	81.05	83.59	86.02	88.35	90.58
15.7	2.67	83.67	0.04	75.60	78.45	81.13	83.67	86.10	88.41	90.62
15.8	2.74	83.75	0.04	75.67	78.53	81.21	83.75	86.17	88.47	90.67
15.9	2.80	83.83	0.04	75.74	78.61	81.29	83.83	86.24	88.53	90.71
16	2.87	83.91	0.04	75.81	78.69	81.37	83.91	86.30	88.58	90.75
16.1	2.94	83.98	0.04	75.88	78.76	81.45	83.98	86.37	88.63	90.79
16.2	3.01	84.05	0.04	75.94	78.84	81.53	84.05	86.43	88.69	90.83
16.3	3.08	84.12	0.04	76.01	78.91	81.60	84.12	86.49	88.74	90.87
16.4	3.15	84.19	0.04	76.07	78.98	81.67	84.19	86.55	88.79	90.90
16.5	3.22	84.26	0.04	76.13	79.05	81.75	84.26	86.61	88.83	90.94
16.6	3.29	84.32	0.04	76.19	79.12	81.82	84.32	86.67	88.88	90.97
16.7	3.37	84.39	0.04	76.25	79.19	81.89	84.39	86.73	88.93	91.00
16.8	3.44	84.45	0.04	76.31	79.26	81.96	84.45	86.78	88.97	91.04
16.9	3.51	84.52	0.04	76.37	79.32	82.02	84.52	86.84	89.02	91.07
17	3.58	84.58	0.04	76.43	79.39	82.09	84.58	86.90	89.06	91.10
17.1	3.65	84.64	0.04	76.49	79.46	82.16	84.64	86.95	89.10	91.13
17.2	3.72	84.71	0.04	76.54	79.52	82.23	84.71	87.00	89.15	91.16
17.3	3.80	84.77	0.04	76.60	79.59	82.29	84.77	87.06	89.19	91.19
17.4	3.87	84.83	0.04	76.66	79.65	82.36	84.83	87.11	89.23	91.22
17.5	3.94	84.89	0.04	76.71	79.72	82.42	84.89	87.16	89.27	91.25
17.6	4.01	84.95	0.04	76.77	79.78	82.49	84.95	87.22	89.32	91.28
17.7	4.08	85.01	0.04	76.82	79.85	82.55	85.01	87.27	89.36	91.31
17.8	4.16	85.07	0.04	76.88	79.91	82.62	85.07	87.32	89.40	91.34
17.9	4.23	85.13	0.04	76.94	79.98	82.69	85.13	87.37	89.44	91.37
18	4.24	85.15	0.04	76.95	79.99	82.70	85.15	87.38	89.45	91.37

**Table 7:** Leg Length in Non-Hispanic White or Mexican American Males.

	LMS Curves			Percentiles						
Age (years)	L	M	S	3rd	10th	25th	50th	75th	90th	97^th^
2	0.09	36.65	0.06	32.72	33.98	35.29	36.65	38.05	39.51	41.01
2.1	0.08	37.13	0.06	33.14	34.43	35.75	37.13	38.55	40.02	41.54
2.2	0.07	37.60	0.06	33.57	34.87	36.21	37.60	39.04	40.53	42.08
2.3	0.07	38.08	0.06	34.00	35.31	36.67	38.08	39.54	41.05	42.61
2.4	0.06	38.56	0.06	34.43	35.76	37.13	38.56	40.03	41.56	43.15
2.5	0.05	39.03	0.06	34.86	36.20	37.59	39.03	40.53	42.07	43.68
2.6	0.05	39.51	0.06	35.28	36.64	38.05	39.51	41.02	42.59	44.21
2.7	0.04	39.98	0.06	35.71	37.08	38.50	39.98	41.51	43.10	44.74
2.8	0.04	40.45	0.06	36.13	37.52	38.96	40.45	42.00	43.61	45.27
2.9	0.03	40.92	0.06	36.55	37.96	39.41	40.92	42.49	44.11	45.80
3	0.03	41.39	0.06	36.97	38.39	39.86	41.39	42.97	44.62	46.32
3.1	0.02	41.86	0.06	37.39	38.82	40.31	41.86	43.46	45.12	46.84
3.2	0.02	42.32	0.06	37.81	39.25	40.76	42.32	43.94	45.62	47.36
3.3	0.01	42.78	0.06	38.22	39.68	41.20	42.78	44.41	46.11	47.87
3.4	0.01	43.23	0.06	38.63	40.11	41.64	43.23	44.89	46.60	48.38
3.5	0.00	43.68	0.06	39.03	40.53	42.08	43.68	45.35	47.09	48.89
3.6	0.00	44.13	0.06	39.44	40.94	42.51	44.13	45.82	47.57	49.39
3.7	0.00	44.58	0.06	39.83	41.36	42.94	44.58	46.28	48.05	49.89
3.8	0.00	45.02	0.06	40.23	41.77	43.36	45.02	46.74	48.52	50.38
3.9	−0.01	45.46	0.06	40.62	42.17	43.78	45.46	47.19	49.00	50.87
4	−0.01	45.89	0.06	41.01	42.57	44.20	45.89	47.64	49.46	51.35
4.1	−0.01	46.32	0.06	41.39	42.97	44.61	46.32	48.09	49.92	51.83
4.2	−0.01	46.74	0.06	41.77	43.37	45.02	46.74	48.53	50.38	52.31
4.3	−0.01	47.16	0.06	42.15	43.76	45.43	47.16	48.97	50.84	52.78
4.4	−0.01	47.58	0.06	42.53	44.15	45.83	47.58	49.40	51.29	53.25
4.5	−0.01	48.00	0.06	42.90	44.53	46.23	48.00	49.83	51.73	53.71
4.6	−0.01	48.41	0.06	43.26	44.91	46.63	48.41	50.26	52.18	54.17
4.7	0.00	48.82	0.06	43.63	45.29	47.02	48.82	50.68	52.62	54.63
4.8	0.00	49.22	0.06	43.99	45.67	47.41	49.22	51.10	53.05	55.08
4.9	0.00	49.62	0.06	44.34	46.04	47.80	49.62	51.52	53.48	55.53
5	0.01	50.02	0.06	44.70	46.41	48.18	50.02	51.93	53.91	55.97
5.1	0.01	50.41	0.06	45.05	46.77	48.56	50.41	52.34	54.34	56.41
5.2	0.02	50.81	0.06	45.40	47.13	48.94	50.81	52.75	54.76	56.85
5.3	0.02	51.20	0.06	45.74	47.49	49.31	51.20	53.15	55.18	57.28
5.4	0.03	51.58	0.06	46.09	47.85	49.68	51.58	53.55	55.60	57.71
5.5	0.03	51.97	0.06	46.43	48.21	50.05	51.97	53.95	56.01	58.14
5.6	0.04	52.35	0.06	46.76	48.56	50.42	52.35	54.35	56.42	58.57
5.7	0.05	52.73	0.06	47.10	48.91	50.78	52.73	54.74	56.83	58.99
5.8	0.05	53.11	0.06	47.43	49.26	51.15	53.11	55.14	57.24	59.42
5.9	0.06	53.48	0.06	47.77	49.60	51.51	53.48	55.53	57.64	59.83
6	0.07	53.86	0.06	48.10	49.95	51.87	53.86	55.91	58.05	60.25
6.1	0.08	54.23	0.06	48.42	50.29	52.23	54.23	56.30	58.45	60.67
6.2	0.09	54.60	0.06	48.75	50.63	52.58	54.60	56.69	58.85	61.08
6.3	0.10	54.97	0.06	49.07	50.97	52.94	54.97	57.07	59.25	61.49
6.4	0.11	55.34	0.06	49.40	51.31	53.29	55.34	57.45	59.64	61.90
6.5	0.12	55.70	0.06	49.72	51.65	53.64	55.70	57.83	60.04	62.31
6.6	0.13	56.06	0.06	50.04	51.98	53.99	56.06	58.21	60.43	62.72
6.7	0.14	56.43	0.06	50.35	52.31	54.34	56.43	58.59	60.82	63.12
6.8	0.15	56.79	0.06	50.67	52.64	54.68	56.79	58.96	61.21	63.52
6.9	0.16	57.14	0.06	50.98	52.97	55.02	57.14	59.33	61.59	63.92
7	0.17	57.50	0.06	51.29	53.30	55.37	57.50	59.70	61.98	64.32
7.1	0.18	57.86	0.06	51.60	53.62	55.70	57.86	60.07	62.36	64.72
7.2	0.19	58.21	0.06	51.91	53.94	56.04	58.21	60.44	62.74	65.11
7.3	0.21	58.56	0.06	52.21	54.26	56.38	58.56	60.80	63.12	65.50
7.4	0.22	58.91	0.06	52.52	54.58	56.71	58.91	61.17	63.50	65.89
7.5	0.23	59.25	0.06	52.82	54.90	57.05	59.25	61.53	63.87	66.28
7.6	0.25	59.60	0.06	53.12	55.21	57.38	59.60	61.89	64.24	66.66
7.7	0.26	59.94	0.06	53.41	55.53	57.70	59.94	62.25	64.61	67.05
7.8	0.27	60.29	0.06	53.71	55.84	58.03	60.29	62.60	64.98	67.43
7.9	0.29	60.63	0.06	54.00	56.15	58.36	60.63	62.96	65.35	67.81
8	0.30	60.96	0.06	54.29	56.46	58.68	60.96	63.31	65.72	68.19
8.1	0.31	61.30	0.06	54.58	56.76	59.00	61.30	63.66	66.08	68.56
8.2	0.33	61.63	0.06	54.87	57.07	59.32	61.63	64.01	66.44	68.93
8.3	0.34	61.97	0.06	55.16	57.37	59.64	61.97	64.35	66.80	69.31
8.4	0.36	62.30	0.06	55.44	57.67	59.96	62.30	64.70	67.16	69.68
8.5	0.37	62.63	0.06	55.73	57.97	60.27	62.63	65.04	67.52	70.05
8.6	0.39	62.96	0.06	56.01	58.27	60.59	62.96	65.39	67.87	70.41
8.7	0.40	63.29	0.06	56.29	58.57	60.90	63.29	65.73	68.22	70.78
8.8	0.42	63.61	0.06	56.57	58.86	61.21	63.61	66.07	68.58	71.14
8.9	0.43	63.93	0.06	56.84	59.15	61.52	63.93	66.40	68.93	71.50
9	0.45	64.26	0.06	57.12	59.45	61.83	64.26	66.74	69.27	71.86
9.1	0.47	64.58	0.06	57.39	59.74	62.13	64.58	67.07	69.62	72.22
9.2	0.48	64.90	0.06	57.66	60.02	62.44	64.90	67.41	69.97	72.58
9.3	0.50	65.21	0.06	57.93	60.31	62.74	65.21	67.74	70.31	72.93
9.4	0.51	65.53	0.06	58.20	60.60	63.04	65.53	68.07	70.65	73.28
9.5	0.53	65.85	0.06	58.47	60.88	63.34	65.85	68.40	70.99	73.63
9.6	0.55	66.16	0.06	58.73	61.16	63.64	66.16	68.72	71.33	73.98
9.7	0.56	66.47	0.06	59.00	61.45	63.94	66.47	69.05	71.67	74.33
9.8	0.58	66.78	0.06	59.26	61.73	64.23	66.78	69.37	72.01	74.68
9.9	0.60	67.09	0.06	59.52	62.01	64.53	67.09	69.70	72.34	75.03
10	0.61	67.40	0.06	59.78	62.28	64.82	67.40	70.02	72.68	75.37
10.1	0.63	67.71	0.06	60.04	62.56	65.12	67.71	70.34	73.01	75.72
10.2	0.65	68.02	0.06	60.30	62.84	65.41	68.02	70.66	73.34	76.06
10.3	0.66	68.32	0.06	60.56	63.11	65.70	68.32	70.98	73.67	76.40
10.4	0.68	68.63	0.06	60.81	63.39	65.99	68.63	71.30	74.01	76.74
10.5	0.70	68.93	0.06	61.07	63.66	66.28	68.93	71.62	74.34	77.08
10.6	0.71	69.24	0.06	61.32	63.93	66.57	69.24	71.94	74.66	77.42
10.7	0.73	69.54	0.06	61.58	64.20	66.86	69.54	72.25	74.99	77.76
10.8	0.74	69.84	0.06	61.83	64.47	67.15	69.84	72.57	75.32	78.10
10.9	0.76	70.15	0.06	62.08	64.75	67.43	70.15	72.88	75.65	78.44
11	0.78	70.45	0.06	62.34	65.02	67.72	70.45	73.20	75.97	78.77
11.1	0.79	70.75	0.06	62.59	65.28	68.00	70.75	73.51	76.30	79.11
11.2	0.81	71.05	0.06	62.84	65.55	68.29	71.05	73.83	76.63	79.44
11.3	0.82	71.35	0.06	63.09	65.82	68.57	71.35	74.14	76.95	79.78
11.4	0.84	71.64	0.06	63.34	66.09	68.86	71.64	74.45	77.27	80.11
11.5	0.85	71.94	0.06	63.58	66.35	69.14	71.94	74.76	77.60	80.45
11.6	0.86	72.24	0.06	63.83	66.62	69.42	72.24	75.07	77.92	80.78
11.7	0.88	72.53	0.06	64.08	66.88	69.70	72.53	75.38	78.24	81.11
11.8	0.89	72.83	0.06	64.32	67.14	69.98	72.83	75.68	78.56	81.44
11.9	0.90	73.12	0.06	64.57	67.41	70.26	73.12	75.99	78.87	81.77
12	0.92	73.41	0.06	64.81	67.66	70.53	73.41	76.29	79.19	82.09
12.1	0.93	73.70	0.06	65.05	67.92	70.81	73.70	76.60	79.50	82.42
12.2	0.94	73.98	0.06	65.29	68.18	71.08	73.98	76.90	79.81	82.74
12.3	0.95	74.27	0.06	65.53	68.43	71.35	74.27	77.19	80.12	83.06
12.4	0.96	74.55	0.06	65.76	68.69	71.62	74.55	77.49	80.43	83.38
12.5	0.97	74.83	0.06	66.00	68.94	71.88	74.83	77.78	80.73	83.69
12.6	0.98	75.11	0.06	66.23	69.19	72.15	75.11	78.07	81.04	84.00
12.7	0.99	75.38	0.06	66.46	69.43	72.41	75.38	78.36	81.34	84.31
12.8	1.00	75.65	0.06	66.68	69.67	72.66	75.65	78.64	81.63	84.62
12.9	1.01	75.92	0.06	66.91	69.92	72.92	75.92	78.92	81.92	84.92
13	1.02	76.19	0.06	67.13	70.15	73.17	76.19	79.20	82.21	85.22
13.1	1.03	76.45	0.06	67.35	70.39	73.42	76.45	79.48	82.50	85.52
13.2	1.04	76.71	0.06	67.57	70.62	73.67	76.71	79.75	82.78	85.81
13.3	1.05	76.97	0.06	67.78	70.85	73.91	76.97	80.02	83.06	86.10
13.4	1.06	77.22	0.06	67.99	71.08	74.15	77.22	80.28	83.34	86.39
13.5	1.06	77.47	0.06	68.20	71.30	74.39	77.47	80.54	83.61	86.67
13.6	1.07	77.71	0.06	68.40	71.52	74.62	77.71	80.80	83.88	86.95
13.7	1.08	77.96	0.06	68.61	71.73	74.85	77.96	81.05	84.14	87.22
13.8	1.08	78.19	0.06	68.80	71.95	75.07	78.19	81.30	84.40	87.49
13.9	1.09	78.43	0.06	69.00	72.15	75.30	78.43	81.55	84.66	87.75
14	1.10	78.66	0.06	69.19	72.36	75.52	78.66	81.79	84.91	88.01
14.1	1.10	78.88	0.06	69.38	72.56	75.73	78.88	82.02	85.15	88.27
14.2	1.11	79.11	0.06	69.57	72.76	75.94	79.11	82.26	85.40	88.52
14.3	1.11	79.32	0.06	69.75	72.96	76.15	79.32	82.49	85.63	88.77
14.4	1.12	79.54	0.06	69.93	73.15	76.35	79.54	82.71	85.87	89.01
14.5	1.12	79.75	0.06	70.10	73.34	76.55	79.75	82.93	86.10	89.25
14.6	1.13	79.96	0.06	70.28	73.52	76.75	79.96	83.15	86.32	89.49
14.7	1.13	80.16	0.06	70.44	73.70	76.94	80.16	83.36	86.55	89.72
14.8	1.14	80.36	0.06	70.61	73.88	77.13	80.36	83.57	86.76	89.94
14.9	1.14	80.55	0.06	70.77	74.05	77.31	80.55	83.77	86.98	90.16
15	1.15	80.74	0.06	70.93	74.22	77.49	80.74	83.97	87.18	90.38
15.1	1.15	80.93	0.06	71.09	74.39	77.67	80.93	84.17	87.39	90.59
15.2	1.16	81.11	0.06	71.24	74.55	77.84	81.11	84.36	87.59	90.80
15.3	1.16	81.29	0.06	71.39	74.71	78.02	81.29	84.55	87.79	91.00
15.4	1.17	81.47	0.06	71.54	74.87	78.18	81.47	84.74	87.98	91.21
15.5	1.17	81.64	0.06	71.68	75.03	78.35	81.64	84.92	88.17	91.40
15.6	1.18	81.81	0.06	71.82	75.18	78.51	81.81	85.10	88.36	91.60
15.7	1.18	81.98	0.06	71.96	75.33	78.67	81.98	85.27	88.54	91.79
15.8	1.18	82.15	0.06	72.10	75.47	78.82	82.15	85.45	88.72	91.97
15.9	1.19	82.31	0.06	72.23	75.62	78.98	82.31	85.62	88.90	92.16
16	1.19	82.47	0.06	72.36	75.76	79.13	82.47	85.78	89.07	92.34
16.1	1.20	82.63	0.06	72.49	75.90	79.28	82.63	85.95	89.25	92.52
16.2	1.20	82.78	0.06	72.62	76.04	79.42	82.78	86.11	89.42	92.70
16.3	1.21	82.93	0.06	72.75	76.17	79.57	82.93	86.27	89.58	92.87
16.4	1.21	83.09	0.06	72.87	76.31	79.71	83.09	86.43	89.75	93.04
16.5	1.22	83.24	0.06	72.99	76.44	79.85	83.24	86.59	89.91	93.21
16.6	1.22	83.38	0.06	73.12	76.57	79.99	83.38	86.74	90.07	93.38
16.7	1.22	83.53	0.06	73.23	76.70	80.13	83.53	86.90	90.24	93.55
16.8	1.23	83.67	0.06	73.35	76.83	80.27	83.67	87.05	90.39	93.71
16.9	1.23	83.82	0.06	73.47	76.96	80.40	83.82	87.20	90.55	93.88
17	1.24	83.96	0.06	73.59	77.08	80.54	83.96	87.35	90.71	94.04
17.1	1.24	84.10	0.06	73.70	77.21	80.67	84.10	87.50	90.87	94.20
17.2	1.25	84.25	0.06	73.82	77.33	80.81	84.25	87.65	91.02	94.36
17.3	1.25	84.39	0.06	73.93	77.46	80.94	84.39	87.80	91.18	94.52
17.4	1.26	84.53	0.06	74.05	77.58	81.07	84.53	87.95	91.33	94.68
17.5	1.26	84.67	0.06	74.16	77.70	81.21	84.67	88.09	91.48	94.84
17.6	1.27	84.81	0.06	74.28	77.83	81.34	84.81	88.24	91.64	95.00
17.7	1.27	84.95	0.06	74.39	77.95	81.47	84.95	88.39	91.79	95.16
17.8	1.28	85.09	0.06	74.50	78.07	81.60	85.09	88.54	91.94	95.32
17.9	1.28	85.23	0.06	74.62	78.20	81.73	85.23	88.68	92.10	95.48
18	1.28	85.26	0.06	74.64	78.22	81.76	85.26	88.71	92.13	95.51

**Table 8:** Leg Length in Non-Hispanic White or Mexican American Females.

	LMS Curves			Percentiles						
Age (years)	L	M	S	3rd	10th	25th	50th	75th	90th	97^th^
2	0.80	36.48	0.06	32.18	33.60	35.03	36.48	37.94	39.41	40.89
2.1	0.79	36.96	0.06	32.61	34.04	35.49	36.96	38.43	39.92	41.42
2.2	0.79	37.43	0.06	33.04	34.49	35.95	37.43	38.92	40.43	41.94
2.3	0.79	37.91	0.06	33.47	34.93	36.41	37.91	39.42	40.93	42.46
2.4	0.79	38.38	0.06	33.89	35.38	36.87	38.38	39.91	41.44	42.99
2.5	0.78	38.86	0.06	34.32	35.82	37.33	38.86	40.40	41.95	43.51
2.6	0.78	39.33	0.06	34.75	36.26	37.79	39.33	40.89	42.45	44.03
2.7	0.78	39.80	0.06	35.18	36.71	38.25	39.80	41.37	42.96	44.55
2.8	0.78	40.27	0.06	35.60	37.15	38.70	40.27	41.86	43.46	45.07
2.9	0.77	40.74	0.06	36.03	37.58	39.16	40.74	42.34	43.96	45.58
3	0.77	41.21	0.06	36.45	38.02	39.61	41.21	42.82	44.45	46.10
3.1	0.77	41.67	0.06	36.87	38.46	40.06	41.67	43.30	44.95	46.61
3.2	0.77	42.13	0.06	37.29	38.89	40.50	42.13	43.78	45.44	47.11
3.3	0.77	42.59	0.06	37.70	39.32	40.95	42.59	44.25	45.93	47.62
3.4	0.76	43.05	0.06	38.12	39.74	41.39	43.05	44.72	46.41	48.11
3.5	0.76	43.50	0.06	38.53	40.17	41.83	43.50	45.19	46.89	48.61
3.6	0.76	43.95	0.06	38.93	40.59	42.26	43.95	45.65	47.37	49.10
3.7	0.76	44.39	0.06	39.34	41.01	42.69	44.39	46.11	47.84	49.59
3.8	0.76	44.84	0.06	39.74	41.42	43.12	44.84	46.57	48.31	50.07
3.9	0.76	45.27	0.06	40.14	41.83	43.55	45.27	47.02	48.78	50.55
4	0.76	45.71	0.06	40.53	42.24	43.97	45.71	47.47	49.24	51.03
4.1	0.76	46.14	0.06	40.92	42.64	44.38	46.14	47.91	49.70	51.50
4.2	0.76	46.57	0.06	41.31	43.05	44.80	46.57	48.35	50.15	51.97
4.3	0.76	46.99	0.06	41.69	43.44	45.21	46.99	48.79	50.60	52.43
4.4	0.76	47.41	0.06	42.08	43.84	45.61	47.41	49.22	51.05	52.89
4.5	0.76	47.83	0.06	42.45	44.23	46.02	47.83	49.65	51.49	53.34
4.6	0.77	48.24	0.06	42.83	44.61	46.42	48.24	50.07	51.93	53.80
4.7	0.77	48.65	0.06	43.20	45.00	46.81	48.65	50.50	52.36	54.24
4.8	0.77	49.05	0.06	43.57	45.38	47.21	49.05	50.92	52.79	54.69
4.9	0.77	49.46	0.06	43.93	45.76	47.60	49.46	51.33	53.22	55.13
5	0.77	49.85	0.06	44.29	46.13	47.98	49.85	51.74	53.65	55.56
5.1	0.77	50.25	0.06	44.65	46.50	48.37	50.25	52.15	54.07	56.00
5.2	0.77	50.64	0.06	45.01	46.87	48.75	50.64	52.56	54.48	56.43
5.3	0.77	51.03	0.06	45.36	47.24	49.13	51.03	52.96	54.90	56.86
5.4	0.77	51.42	0.06	45.71	47.60	49.50	51.42	53.36	55.31	57.28
5.5	0.77	51.81	0.06	46.06	47.96	49.87	51.81	53.75	55.72	57.70
5.6	0.77	52.19	0.06	46.41	48.32	50.24	52.19	54.15	56.13	58.12
5.7	0.77	52.57	0.06	46.75	48.67	50.61	52.57	54.54	56.53	58.54
5.8	0.77	52.94	0.06	47.09	49.03	50.98	52.94	54.93	56.93	58.95
5.9	0.77	53.32	0.06	47.43	49.38	51.34	53.32	55.32	57.33	59.36
6	0.77	53.69	0.06	47.77	49.73	51.70	53.69	55.70	57.73	59.77
6.1	0.77	54.06	0.06	48.10	50.07	52.06	54.06	56.08	58.12	60.18
6.2	0.77	54.43	0.06	48.44	50.42	52.41	54.43	56.46	58.51	60.58
6.3	0.77	54.80	0.06	48.77	50.76	52.77	54.80	56.84	58.90	60.98
6.4	0.76	55.16	0.06	49.10	51.10	53.12	55.16	57.22	59.29	61.38
6.5	0.76	55.52	0.06	49.42	51.44	53.47	55.52	57.59	59.68	61.78
6.6	0.76	55.88	0.06	49.75	51.78	53.82	55.88	57.96	60.06	62.18
6.7	0.76	56.24	0.06	50.07	52.11	54.17	56.24	58.33	60.45	62.57
6.8	0.76	56.60	0.06	50.40	52.45	54.51	56.60	58.70	60.83	62.97
6.9	0.76	56.96	0.06	50.72	52.78	54.86	56.96	59.07	61.21	63.36
7	0.76	57.31	0.06	51.04	53.11	55.20	57.31	59.44	61.59	63.75
7.1	0.76	57.66	0.06	51.36	53.44	55.54	57.66	59.80	61.96	64.14
7.2	0.76	58.02	0.06	51.67	53.77	55.88	58.02	60.17	62.34	64.53
7.3	0.76	58.37	0.06	51.99	54.10	56.22	58.37	60.53	62.71	64.92
7.4	0.76	58.72	0.06	52.31	54.42	56.56	58.72	60.89	63.09	65.30
7.5	0.75	59.06	0.06	52.62	54.75	56.90	59.06	61.25	63.46	65.69
7.6	0.75	59.41	0.06	52.93	55.07	57.23	59.41	61.61	63.83	66.07
7.7	0.75	59.76	0.06	53.25	55.39	57.57	59.76	61.97	64.20	66.45
7.8	0.75	60.10	0.06	53.56	55.72	57.90	60.10	62.33	64.57	66.83
7.9	0.75	60.45	0.06	53.87	56.04	58.23	60.45	62.68	64.94	67.21
8	0.75	60.79	0.06	54.17	56.36	58.56	60.79	63.03	65.30	67.59
8.1	0.75	61.13	0.06	54.48	56.67	58.89	61.13	63.39	65.67	67.96
8.2	0.75	61.47	0.06	54.79	56.99	59.22	61.47	63.74	66.03	68.34
8.3	0.75	61.81	0.06	55.09	57.31	59.55	61.81	64.09	66.39	68.71
8.4	0.75	62.14	0.06	55.39	57.62	59.87	62.14	64.44	66.75	69.08
8.5	0.75	62.48	0.06	55.69	57.93	60.20	62.48	64.78	67.11	69.45
8.6	0.75	62.81	0.06	55.99	58.25	60.52	62.81	65.13	67.47	69.82
8.7	0.75	63.14	0.06	56.29	58.56	60.84	63.14	65.47	67.82	70.19
8.8	0.75	63.48	0.06	56.59	58.86	61.16	63.48	65.81	68.17	70.56
8.9	0.75	63.80	0.05	56.89	59.17	61.48	63.80	66.15	68.53	70.92
9	0.75	64.13	0.05	57.18	59.48	61.79	64.13	66.49	68.88	71.28
9.1	0.75	64.46	0.05	57.47	59.78	62.11	64.46	66.83	69.22	71.64
9.2	0.75	64.78	0.05	57.76	60.08	62.42	64.78	67.17	69.57	72.00
9.3	0.75	65.10	0.05	58.05	60.38	62.73	65.10	67.50	69.92	72.35
9.4	0.75	65.42	0.05	58.34	60.68	63.04	65.42	67.83	70.26	72.71
9.5	0.75	65.74	0.05	58.62	60.97	63.35	65.74	68.16	70.60	73.06
9.6	0.76	66.06	0.05	58.91	61.27	63.65	66.06	68.49	70.94	73.41
9.7	0.76	66.37	0.05	59.19	61.56	63.95	66.37	68.81	71.27	73.75
9.8	0.76	66.68	0.05	59.46	61.85	64.25	66.68	69.13	71.60	74.09
9.9	0.76	66.99	0.05	59.74	62.13	64.55	66.99	69.45	71.93	74.43
10	0.77	67.29	0.05	60.01	62.42	64.84	67.29	69.77	72.26	74.77
10.1	0.77	67.60	0.05	60.28	62.70	65.13	67.60	70.08	72.58	75.10
10.2	0.77	67.89	0.05	60.54	62.97	65.42	67.89	70.39	72.90	75.43
10.3	0.78	68.19	0.05	60.80	63.24	65.71	68.19	70.69	73.21	75.75
10.4	0.78	68.48	0.05	61.06	63.51	65.99	68.48	70.99	73.52	76.07
10.5	0.79	68.77	0.05	61.32	63.78	66.26	68.77	71.29	73.83	76.39
10.6	0.79	69.05	0.05	61.57	64.04	66.53	69.05	71.58	74.13	76.70
10.7	0.80	69.32	0.05	61.81	64.30	66.80	69.32	71.87	74.43	77.01
10.8	0.80	69.60	0.05	62.06	64.55	67.06	69.60	72.15	74.72	77.31
10.9	0.81	69.87	0.05	62.29	64.80	67.32	69.87	72.43	75.00	77.60
11	0.81	70.13	0.05	62.52	65.04	67.58	70.13	72.70	75.29	77.89
11.1	0.82	70.39	0.05	62.75	65.28	67.82	70.39	72.96	75.56	78.17
11.2	0.83	70.64	0.05	62.97	65.51	68.07	70.64	73.22	75.83	78.45
11.3	0.83	70.88	0.05	63.19	65.74	68.30	70.88	73.48	76.09	78.72
11.4	0.84	71.12	0.05	63.40	65.96	68.53	71.12	73.73	76.35	78.98
11.5	0.85	71.35	0.05	63.60	66.17	68.76	71.35	73.97	76.59	79.24
11.6	0.86	71.58	0.05	63.80	66.38	68.97	71.58	74.20	76.84	79.48
11.7	0.87	71.80	0.05	64.00	66.58	69.19	71.80	74.43	77.07	79.72
11.8	0.88	72.02	0.05	64.18	66.78	69.39	72.02	74.65	77.30	79.96
11.9	0.89	72.22	0.05	64.36	66.97	69.59	72.22	74.87	77.52	80.19
12	0.90	72.43	0.05	64.54	67.16	69.79	72.43	75.08	77.74	80.40
12.1	0.91	72.62	0.05	64.71	67.34	69.97	72.62	75.28	77.94	80.62
12.2	0.92	72.81	0.05	64.87	67.51	70.16	72.81	75.47	78.14	80.82
12.3	0.93	72.99	0.05	65.03	67.68	70.33	72.99	75.66	78.34	81.02
12.4	0.94	73.17	0.05	65.18	67.84	70.50	73.17	75.84	78.52	81.21
12.5	0.96	73.34	0.05	65.32	67.99	70.66	73.34	76.02	78.70	81.39
12.6	0.97	73.50	0.05	65.46	68.14	70.82	73.50	76.19	78.88	81.57
12.7	0.98	73.66	0.05	65.60	68.28	70.97	73.66	76.35	79.04	81.74
12.8	1.00	73.81	0.05	65.73	68.42	71.12	73.81	76.51	79.20	81.90
12.9	1.01	73.96	0.05	65.85	68.55	71.26	73.96	76.66	79.36	82.05
13	1.03	74.10	0.05	65.97	68.68	71.39	74.10	76.80	79.50	82.20
13.1	1.05	74.23	0.05	66.08	68.80	71.52	74.23	76.94	79.65	82.35
13.2	1.06	74.36	0.05	66.18	68.92	71.64	74.36	77.07	79.78	82.48
13.3	1.08	74.48	0.05	66.29	69.03	71.76	74.48	77.20	79.91	82.61
13.4	1.10	74.60	0.05	66.38	69.13	71.87	74.60	77.32	80.03	82.73
13.5	1.12	74.72	0.05	66.48	69.23	71.98	74.72	77.44	80.15	82.85
13.6	1.13	74.82	0.05	66.56	69.33	72.08	74.82	77.55	80.26	82.96
13.7	1.15	74.93	0.05	66.65	69.42	72.18	74.93	77.66	80.37	83.07
13.8	1.17	75.03	0.05	66.72	69.51	72.28	75.03	77.76	80.47	83.17
13.9	1.19	75.12	0.05	66.80	69.59	72.37	75.12	77.85	80.57	83.27
14	1.21	75.21	0.05	66.87	69.67	72.45	75.21	77.95	80.66	83.36
14.1	1.24	75.30	0.05	66.93	69.75	72.53	75.30	78.03	80.75	83.44
14.2	1.26	75.38	0.05	66.99	69.82	72.61	75.38	78.12	80.83	83.53
14.3	1.28	75.45	0.05	67.05	69.88	72.68	75.45	78.20	80.91	83.60
14.4	1.30	75.53	0.05	67.11	69.95	72.75	75.53	78.27	80.99	83.67
14.5	1.32	75.60	0.05	67.16	70.01	72.82	75.60	78.34	81.06	83.74
14.6	1.35	75.67	0.05	67.21	70.07	72.88	75.67	78.41	81.13	83.81
14.7	1.37	75.73	0.05	67.25	70.12	72.94	75.73	78.48	81.19	83.87
14.8	1.39	75.79	0.05	67.29	70.17	73.00	75.79	78.54	81.25	83.93
14.9	1.42	75.85	0.05	67.33	70.22	73.05	75.85	78.60	81.31	83.98
15	1.44	75.90	0.05	67.37	70.26	73.11	75.90	78.65	81.36	84.03
15.1	1.47	75.95	0.05	67.40	70.31	73.15	75.95	78.70	81.41	84.08
15.2	1.49	76.00	0.05	67.43	70.35	73.20	76.00	78.75	81.46	84.12
15.3	1.52	76.05	0.05	67.46	70.38	73.24	76.05	78.80	81.51	84.16
15.4	1.54	76.09	0.05	67.49	70.42	73.29	76.09	78.85	81.55	84.20
15.5	1.57	76.14	0.05	67.51	70.45	73.33	76.14	78.89	81.59	84.24
15.6	1.59	76.18	0.05	67.53	70.48	73.36	76.18	78.93	81.63	84.28
15.7	1.62	76.22	0.05	67.56	70.52	73.40	76.22	78.97	81.67	84.31
15.8	1.64	76.25	0.05	67.57	70.54	73.43	76.25	79.01	81.70	84.34
15.9	1.67	76.29	0.05	67.59	70.57	73.47	76.29	79.05	81.74	84.37
16	1.69	76.32	0.05	67.61	70.60	73.50	76.32	79.08	81.77	84.40
16.1	1.72	76.36	0.05	67.62	70.62	73.53	76.36	79.11	81.80	84.43
16.2	1.74	76.39	0.05	67.64	70.64	73.56	76.39	79.15	81.83	84.45
16.3	1.77	76.42	0.05	67.65	70.67	73.59	76.42	79.18	81.86	84.48
16.4	1.79	76.45	0.05	67.66	70.69	73.61	76.45	79.21	81.89	84.50
16.5	1.82	76.48	0.05	67.67	70.71	73.64	76.48	79.24	81.92	84.52
16.6	1.85	76.51	0.05	67.68	70.73	73.67	76.51	79.26	81.94	84.55
16.7	1.87	76.54	0.05	67.69	70.75	73.69	76.54	79.29	81.97	84.57
16.8	1.90	76.57	0.05	67.70	70.77	73.72	76.57	79.32	81.99	84.59
16.9	1.93	76.59	0.05	67.71	70.79	73.74	76.59	79.35	82.02	84.61
17	1.95	76.62	0.05	67.72	70.80	73.77	76.62	79.37	82.04	84.63
17.1	1.98	76.65	0.05	67.73	70.82	73.79	76.65	79.40	82.07	84.65
17.2	2.01	76.67	0.05	67.73	70.84	73.81	76.67	79.43	82.09	84.67
17.3	2.03	76.70	0.05	67.74	70.86	73.84	76.70	79.45	82.11	84.69
17.4	2.06	76.72	0.05	67.74	70.87	73.86	76.72	79.48	82.14	84.71
17.5	2.09	76.75	0.05	67.75	70.89	73.88	76.75	79.50	82.16	84.72
17.6	2.11	76.77	0.05	67.76	70.91	73.90	76.77	79.53	82.18	84.74
17.7	2.14	76.80	0.05	67.76	70.92	73.93	76.80	79.55	82.20	84.76
17.8	2.17	76.83	0.05	67.77	70.94	73.95	76.83	79.58	82.23	84.78
17.9	2.20	76.85	0.05	67.77	70.95	73.97	76.85	79.61	82.25	84.80
18	2.20	76.86	0.05	67.77	70.96	73.98	76.86	79.61	82.26	84.80

**Table 9:** Leg Length in Non-Hispanic Black Males.

	LMS Curves			Percentiles						
Age (years)	L	M	S	3rd	10th	25th	50th	75th	90th	97^th^
2	0.84	37.59	0.05	33.61	34.93	36.26	37.59	38.94	40.29	41.65
2.1	0.83	38.13	0.05	34.09	35.43	36.77	38.13	39.50	40.87	42.25
2.2	0.83	38.67	0.05	34.56	35.92	37.29	38.67	40.05	41.45	42.85
2.3	0.82	39.21	0.05	35.04	36.42	37.81	39.21	40.61	42.03	43.45
2.4	0.81	39.74	0.05	35.52	36.92	38.33	39.74	41.17	42.61	44.05
2.5	0.81	40.28	0.05	36.00	37.41	38.84	40.28	41.73	43.18	44.65
2.6	0.80	40.81	0.05	36.47	37.91	39.36	40.81	42.28	43.76	45.25
2.7	0.80	41.35	0.05	36.94	38.40	39.87	41.35	42.83	44.33	45.84
2.8	0.79	41.88	0.05	37.42	38.89	40.38	41.88	43.38	44.91	46.44
2.9	0.79	42.40	0.05	37.88	39.38	40.88	42.40	43.93	45.47	47.03
3	0.78	42.93	0.05	38.35	39.86	41.39	42.93	44.48	46.04	47.61
3.1	0.78	43.45	0.05	38.81	40.34	41.89	43.45	45.02	46.60	48.20
3.2	0.77	43.96	0.05	39.27	40.82	42.39	43.96	45.55	47.16	48.78
3.3	0.77	44.48	0.05	39.72	41.29	42.88	44.48	46.09	47.71	49.35
3.4	0.77	44.98	0.05	40.17	41.76	43.37	44.98	46.61	48.26	49.92
3.5	0.76	45.49	0.05	40.62	42.23	43.85	45.49	47.14	48.80	50.48
3.6	0.76	45.98	0.05	41.06	42.68	44.33	45.98	47.66	49.34	51.04
3.7	0.76	46.48	0.05	41.49	43.14	44.80	46.48	48.17	49.87	51.59
3.8	0.76	46.96	0.05	41.92	43.59	45.27	46.96	48.68	50.40	52.14
3.9	0.76	47.45	0.05	42.35	44.03	45.73	47.45	49.18	50.92	52.68
4	0.75	47.93	0.05	42.77	44.47	46.19	47.93	49.67	51.44	53.22
4.1	0.75	48.40	0.05	43.19	44.91	46.64	48.40	50.17	51.95	53.75
4.2	0.75	48.86	0.05	43.60	45.34	47.09	48.86	50.65	52.46	54.27
4.3	0.76	49.33	0.05	44.00	45.76	47.54	49.33	51.13	52.96	54.79
4.4	0.76	49.78	0.05	44.41	46.18	47.98	49.78	51.61	53.45	55.31
4.5	0.76	50.24	0.05	44.80	46.60	48.41	50.24	52.08	53.94	55.82
4.6	0.76	50.68	0.05	45.20	47.01	48.84	50.68	52.55	54.43	56.32
4.7	0.76	51.13	0.05	45.58	47.41	49.26	51.13	53.01	54.91	56.82
4.8	0.76	51.57	0.06	45.97	47.82	49.68	51.57	53.47	55.38	57.31
4.9	0.76	52.00	0.06	46.34	48.21	50.10	52.00	53.92	55.85	57.80
5	0.77	52.43	0.06	46.72	48.60	50.51	52.43	54.36	56.32	58.28
5.1	0.77	52.85	0.06	47.09	48.99	50.91	52.85	54.80	56.78	58.76
5.2	0.77	53.27	0.06	47.45	49.37	51.31	53.27	55.24	57.23	59.23
5.3	0.78	53.68	0.06	47.81	49.75	51.71	53.68	55.67	57.68	59.70
5.4	0.78	54.09	0.06	48.17	50.13	52.10	54.09	56.10	58.13	60.17
5.5	0.78	54.50	0.06	48.52	50.49	52.49	54.50	56.52	58.57	60.62
5.6	0.79	54.90	0.06	48.86	50.86	52.87	54.90	56.94	59.00	61.08
5.7	0.79	55.30	0.06	49.21	51.22	53.25	55.30	57.36	59.43	61.53
5.8	0.80	55.69	0.06	49.55	51.58	53.63	55.69	57.77	59.86	61.97
5.9	0.80	56.08	0.06	49.88	51.93	54.00	56.08	58.17	60.29	62.41
6	0.81	56.46	0.06	50.21	52.28	54.36	56.46	58.58	60.71	62.85
6.1	0.81	56.84	0.06	50.54	52.63	54.73	56.84	58.98	61.12	63.28
6.2	0.82	57.22	0.06	50.87	52.97	55.09	57.22	59.37	61.54	63.71
6.3	0.82	57.60	0.06	51.19	53.31	55.45	57.60	59.77	61.95	64.14
6.4	0.83	57.97	0.06	51.51	53.65	55.80	57.97	60.16	62.35	64.56
6.5	0.83	58.34	0.06	51.83	53.98	56.16	58.34	60.54	62.76	64.98
6.6	0.84	58.71	0.06	52.14	54.32	56.51	58.71	60.93	63.16	65.40
6.7	0.85	59.08	0.06	52.45	54.65	56.86	59.08	61.31	63.56	65.82
6.8	0.85	59.44	0.06	52.76	54.98	57.20	59.44	61.69	63.96	66.23
6.9	0.86	59.80	0.06	53.07	55.30	57.55	59.80	62.07	64.35	66.65
7	0.87	60.16	0.06	53.38	55.63	57.89	60.16	62.45	64.75	67.06
7.1	0.87	60.52	0.06	53.68	55.95	58.23	60.52	62.82	65.14	67.46
7.2	0.88	60.88	0.06	53.98	56.27	58.57	60.88	63.20	65.53	67.87
7.3	0.89	61.23	0.06	54.28	56.59	58.90	61.23	63.57	65.92	68.27
7.4	0.89	61.59	0.06	54.58	56.90	59.24	61.59	63.94	66.30	68.68
7.5	0.90	61.94	0.06	54.88	57.22	59.57	61.94	64.31	66.69	69.08
7.6	0.91	62.29	0.06	55.17	57.54	59.91	62.29	64.68	67.08	69.48
7.7	0.91	62.64	0.06	55.47	57.85	60.24	62.64	65.05	67.46	69.88
7.8	0.92	62.99	0.06	55.76	58.16	60.57	62.99	65.41	67.84	70.28
7.9	0.93	63.34	0.06	56.05	58.47	60.90	63.34	65.78	68.23	70.68
8	0.93	63.68	0.06	56.35	58.79	61.23	63.68	66.14	68.61	71.08
8.1	0.94	64.03	0.06	56.64	59.10	61.56	64.03	66.51	68.99	71.48
8.2	0.95	64.38	0.06	56.93	59.41	61.89	64.38	66.87	69.37	71.87
8.3	0.95	64.72	0.06	57.22	59.72	62.22	64.72	67.24	69.75	72.27
8.4	0.96	65.07	0.06	57.51	60.03	62.55	65.07	67.60	70.13	72.67
8.5	0.96	65.42	0.06	57.80	60.34	62.87	65.42	67.96	70.51	73.07
8.6	0.97	65.76	0.06	58.09	60.64	63.20	65.76	68.33	70.90	73.47
8.7	0.97	66.11	0.06	58.38	60.95	63.53	66.11	68.69	71.28	73.87
8.8	0.97	66.45	0.06	58.67	61.26	63.86	66.45	69.06	71.66	74.26
8.9	0.98	66.80	0.06	58.96	61.57	64.18	66.80	69.42	72.04	74.66
9	0.98	67.15	0.06	59.25	61.88	64.51	67.15	69.78	72.42	75.06
9.1	0.98	67.49	0.06	59.54	62.19	64.84	67.49	70.15	72.80	75.46
9.2	0.98	67.84	0.06	59.83	62.50	65.16	67.84	70.51	73.18	75.86
9.3	0.98	68.18	0.06	60.12	62.80	65.49	68.18	70.87	73.56	76.26
9.4	0.98	68.53	0.06	60.41	63.11	65.82	68.53	71.23	73.95	76.66
9.5	0.99	68.87	0.06	60.70	63.42	66.14	68.87	71.60	74.33	77.06
9.6	0.99	69.21	0.06	60.98	63.73	66.47	69.21	71.96	74.71	77.45
9.7	0.98	69.56	0.06	61.27	64.03	66.79	69.56	72.32	75.09	77.85
9.8	0.98	69.90	0.06	61.56	64.34	67.12	69.90	72.68	75.46	78.25
9.9	0.98	70.24	0.06	61.85	64.64	67.44	70.24	73.04	75.84	78.65
10	0.98	70.58	0.06	62.13	64.95	67.76	70.58	73.40	76.22	79.04
10.1	0.98	70.92	0.06	62.42	65.25	68.08	70.92	73.75	76.59	79.43
10.2	0.97	71.25	0.06	62.71	65.55	68.40	71.25	74.11	76.97	79.83
10.3	0.97	71.59	0.06	62.99	65.85	68.72	71.59	74.46	77.34	80.22
10.4	0.97	71.93	0.06	63.28	66.16	69.04	71.93	74.82	77.71	80.61
10.5	0.96	72.26	0.06	63.56	66.45	69.36	72.26	75.17	78.08	81.00
10.6	0.96	72.59	0.06	63.84	66.75	69.67	72.59	75.52	78.45	81.39
10.7	0.96	72.92	0.06	64.12	67.05	69.98	72.92	75.87	78.82	81.77
10.8	0.95	73.25	0.06	64.40	67.34	70.29	73.25	76.21	79.18	82.16
10.9	0.95	73.58	0.06	64.68	67.64	70.60	73.58	76.56	79.54	82.54
11	0.94	73.90	0.06	64.95	67.93	70.91	73.90	76.90	79.90	82.92
11.1	0.94	74.22	0.06	65.23	68.22	71.22	74.22	77.24	80.26	83.29
11.2	0.93	74.54	0.06	65.50	68.51	71.52	74.54	77.58	80.62	83.66
11.3	0.93	74.86	0.06	65.77	68.79	71.82	74.86	77.91	80.97	84.04
11.4	0.92	75.18	0.06	66.04	69.08	72.12	75.18	78.24	81.32	84.40
11.5	0.92	75.49	0.06	66.31	69.36	72.42	75.49	78.57	81.66	84.77
11.6	0.91	75.80	0.06	66.57	69.64	72.71	75.80	78.90	82.01	85.13
11.7	0.91	76.11	0.06	66.83	69.91	73.00	76.11	79.22	82.35	85.49
11.8	0.90	76.41	0.06	67.09	70.19	73.29	76.41	79.54	82.69	85.84
11.9	0.90	76.71	0.06	67.35	70.46	73.58	76.71	79.86	83.02	86.19
12	0.90	77.01	0.06	67.60	70.73	73.86	77.01	80.17	83.35	86.54
12.1	0.89	77.31	0.06	67.86	70.99	74.14	77.31	80.48	83.68	86.88
12.2	0.89	77.60	0.06	68.11	71.25	74.42	77.60	80.79	84.00	87.22
12.3	0.89	77.89	0.06	68.35	71.52	74.69	77.89	81.10	84.32	87.55
12.4	0.89	78.17	0.06	68.60	71.77	74.97	78.17	81.40	84.63	87.89
12.5	0.88	78.46	0.06	68.84	72.03	75.23	78.46	81.69	84.95	88.21
12.6	0.88	78.74	0.06	69.08	72.28	75.50	78.74	81.99	85.25	88.53
12.7	0.88	79.01	0.06	69.31	72.53	75.76	79.01	82.28	85.56	88.85
12.8	0.88	79.28	0.06	69.54	72.77	76.02	79.28	82.56	85.85	89.16
12.9	0.88	79.55	0.06	69.77	73.01	76.27	79.55	82.84	86.15	89.47
13	0.89	79.81	0.06	69.99	73.25	76.52	79.81	83.12	86.44	89.77
13.1	0.89	80.07	0.06	70.22	73.49	76.77	80.07	83.39	86.72	90.07
13.2	0.89	80.33	0.06	70.43	73.72	77.01	80.33	83.66	87.00	90.36
13.3	0.89	80.58	0.06	70.65	73.94	77.25	80.58	83.92	87.28	90.65
13.4	0.90	80.83	0.06	70.85	74.16	77.49	80.83	84.18	87.54	90.92
13.5	0.90	81.07	0.06	71.06	74.38	77.72	81.07	84.43	87.81	91.20
13.6	0.91	81.31	0.06	71.26	74.60	77.94	81.31	84.68	88.07	91.47
13.7	0.91	81.54	0.06	71.46	74.80	78.17	81.54	84.92	88.32	91.73
13.8	0.92	81.77	0.06	71.65	75.01	78.38	81.77	85.16	88.57	91.98
13.9	0.93	81.99	0.06	71.84	75.21	78.60	81.99	85.40	88.81	92.24
14	0.94	82.21	0.06	72.02	75.41	78.81	82.21	85.63	89.05	92.48
14.1	0.95	82.43	0.06	72.20	75.60	79.01	82.43	85.85	89.28	92.72
14.2	0.96	82.64	0.06	72.38	75.79	79.21	82.64	86.07	89.51	92.95
14.3	0.97	82.84	0.06	72.55	75.98	79.41	82.84	86.29	89.73	93.18
14.4	0.98	83.05	0.06	72.72	76.16	79.60	83.05	86.50	89.95	93.40
14.5	0.99	83.24	0.06	72.88	76.33	79.79	83.24	86.70	90.16	93.62
14.6	1.01	83.44	0.06	73.04	76.50	79.97	83.44	86.90	90.36	93.83
14.7	1.02	83.62	0.06	73.19	76.67	80.15	83.62	87.10	90.56	94.03
14.8	1.04	83.81	0.06	73.34	76.83	80.32	83.81	87.29	90.76	94.23
14.9	1.06	83.99	0.06	73.48	76.99	80.49	83.99	87.47	90.95	94.42
15	1.07	84.16	0.06	73.62	77.15	80.66	84.16	87.65	91.14	94.61
15.1	1.09	84.33	0.06	73.76	77.30	80.82	84.33	87.83	91.32	94.79
15.2	1.11	84.50	0.06	73.89	77.45	80.98	84.50	88.00	91.49	94.97
15.3	1.13	84.66	0.06	74.02	77.59	81.14	84.66	88.17	91.66	95.14
15.4	1.15	84.82	0.06	74.14	77.73	81.29	84.82	88.34	91.83	95.30
15.5	1.17	84.98	0.06	74.26	77.86	81.43	84.98	88.50	91.99	95.47
15.6	1.19	85.13	0.06	74.38	78.00	81.58	85.13	88.65	92.15	95.62
15.7	1.22	85.28	0.06	74.50	78.13	81.72	85.28	88.81	92.31	95.78
15.8	1.24	85.43	0.06	74.61	78.25	81.86	85.43	88.96	92.46	95.93
15.9	1.26	85.57	0.06	74.71	78.37	81.99	85.57	89.11	92.61	96.07
16	1.29	85.71	0.06	74.82	78.50	82.12	85.71	89.25	92.75	96.21
16.1	1.31	85.85	0.06	74.92	78.61	82.25	85.85	89.39	92.89	96.35
16.2	1.34	85.98	0.06	75.02	78.73	82.38	85.98	89.53	93.03	96.49
16.3	1.36	86.11	0.06	75.12	78.84	82.51	86.11	89.67	93.17	96.62
16.4	1.39	86.25	0.06	75.21	78.95	82.63	86.25	89.80	93.30	96.75
16.5	1.42	86.37	0.06	75.30	79.06	82.75	86.37	89.93	93.43	96.88
16.6	1.44	86.50	0.06	75.39	79.17	82.87	86.50	90.06	93.56	97.01
16.7	1.47	86.63	0.06	75.48	79.28	82.99	86.63	90.19	93.69	97.13
16.8	1.50	86.75	0.06	75.57	79.39	83.11	86.75	90.32	93.82	97.26
16.9	1.53	86.88	0.06	75.66	79.49	83.23	86.88	90.45	93.95	97.38
17	1.55	87.00	0.06	75.75	79.60	83.34	87.00	90.58	94.07	97.50
17.1	1.58	87.12	0.06	75.83	79.70	83.46	87.12	90.70	94.20	97.62
17.2	1.61	87.25	0.06	75.92	79.80	83.57	87.25	90.83	94.32	97.74
17.3	1.64	87.37	0.06	76.00	79.90	83.69	87.37	90.95	94.45	97.86
17.4	1.66	87.49	0.06	76.09	80.01	83.80	87.49	91.07	94.57	97.98
17.5	1.69	87.61	0.06	76.17	80.11	83.92	87.61	91.20	94.69	98.10
17.6	1.72	87.73	0.06	76.25	80.21	84.03	87.73	91.32	94.81	98.22
17.7	1.75	87.85	0.06	76.33	80.31	84.14	87.85	91.45	94.94	98.34
17.8	1.78	87.97	0.06	76.42	80.41	84.26	87.97	91.57	95.06	98.45
17.9	1.80	88.09	0.06	76.50	80.51	84.37	88.09	91.69	95.18	98.57
18	1.81	88.12	0.06	76.52	80.53	84.39	88.12	91.72	95.21	98.60

**Table 10:** Leg Length in Non-Hispanic Black Females.

	LMS Curves			Percentiles						
Age (years)	L	M	S	3rd	10th	25th	50th	75th	90th	97^th^
2	0.35	37.86	0.05	33.93	35.21	36.52	37.86	39.24	40.64	42.08
2.1	0.35	38.37	0.05	34.37	35.67	37.00	38.37	39.76	41.19	42.64
2.2	0.35	38.87	0.05	34.82	36.14	37.49	38.87	40.28	41.73	43.21
2.3	0.36	39.37	0.05	35.27	36.61	37.97	39.37	40.81	42.27	43.77
2.4	0.36	39.88	0.05	35.72	37.07	38.46	39.88	41.33	42.82	44.33
2.5	0.36	40.38	0.05	36.17	37.54	38.94	40.38	41.85	43.36	44.90
2.6	0.37	40.88	0.05	36.61	38.00	39.43	40.88	42.38	43.90	45.46
2.7	0.37	41.39	0.05	37.06	38.47	39.91	41.39	42.90	44.44	46.02
2.8	0.37	41.89	0.05	37.51	38.93	40.39	41.89	43.42	44.98	46.58
2.9	0.37	42.39	0.05	37.95	39.39	40.87	42.39	43.94	45.52	47.14
3	0.38	42.89	0.05	38.39	39.86	41.35	42.89	44.45	46.06	47.70
3.1	0.38	43.38	0.05	38.83	40.32	41.83	43.38	44.97	46.59	48.25
3.2	0.38	43.88	0.05	39.27	40.77	42.31	43.88	45.49	47.13	48.81
3.3	0.38	44.37	0.05	39.71	41.23	42.78	44.37	46.00	47.66	49.36
3.4	0.38	44.86	0.05	40.15	41.68	43.26	44.86	46.51	48.19	49.91
3.5	0.38	45.35	0.05	40.58	42.14	43.73	45.35	47.02	48.72	50.46
3.6	0.39	45.84	0.05	41.01	42.59	44.20	45.84	47.52	49.24	51.00
3.7	0.39	46.32	0.05	41.44	43.03	44.66	46.32	48.03	49.77	51.54
3.8	0.39	46.81	0.05	41.87	43.48	45.12	46.81	48.53	50.29	52.08
3.9	0.38	47.28	0.05	42.30	43.92	45.58	47.28	49.02	50.80	52.62
4	0.38	47.76	0.05	42.72	44.36	46.04	47.76	49.52	51.31	53.15
4.1	0.38	48.23	0.05	43.14	44.79	46.49	48.23	50.01	51.82	53.68
4.2	0.38	48.70	0.05	43.55	45.23	46.94	48.70	50.49	52.33	54.21
4.3	0.38	49.16	0.05	43.96	45.66	47.39	49.16	50.98	52.83	54.73
4.4	0.38	49.63	0.05	44.37	46.09	47.84	49.63	51.46	53.33	55.25
4.5	0.37	50.09	0.05	44.78	46.51	48.28	50.09	51.94	53.83	55.77
4.6	0.37	50.54	0.05	45.19	46.93	48.72	50.54	52.41	54.33	56.28
4.7	0.37	51.00	0.05	45.59	47.35	49.15	51.00	52.89	54.82	56.79
4.8	0.36	51.45	0.05	45.99	47.77	49.59	51.45	53.35	55.31	57.30
4.9	0.36	51.90	0.05	46.39	48.18	50.02	51.90	53.82	55.79	57.81
5	0.35	52.34	0.06	46.78	48.59	50.44	52.34	54.28	56.27	58.31
5.1	0.34	52.78	0.06	47.18	49.00	50.87	52.78	54.75	56.75	58.81
5.2	0.34	53.22	0.06	47.57	49.40	51.29	53.22	55.20	57.23	59.31
5.3	0.33	53.66	0.06	47.95	49.81	51.71	53.66	55.66	57.70	59.80
5.4	0.32	54.09	0.06	48.34	50.21	52.12	54.09	56.11	58.17	60.29
5.5	0.32	54.52	0.06	48.72	50.60	52.54	54.52	56.56	58.64	60.78
5.6	0.31	54.95	0.06	49.10	51.00	52.95	54.95	57.00	59.11	61.26
5.7	0.30	55.37	0.06	49.47	51.39	53.35	55.37	57.44	59.57	61.74
5.8	0.29	55.79	0.06	49.85	51.78	53.76	55.79	57.88	60.02	62.22
5.9	0.28	56.21	0.06	50.22	52.16	54.16	56.21	58.32	60.48	62.70
6	0.27	56.62	0.06	50.59	52.54	54.56	56.62	58.75	60.93	63.17
6.1	0.26	57.03	0.06	50.95	52.92	54.95	57.03	59.18	61.38	63.64
6.2	0.25	57.44	0.06	51.32	53.30	55.34	57.44	59.60	61.82	64.10
6.3	0.24	57.85	0.06	51.68	53.68	55.73	57.85	60.03	62.27	64.57
6.4	0.23	58.25	0.06	52.04	54.05	56.12	58.25	60.45	62.71	65.03
6.5	0.22	58.66	0.06	52.39	54.42	56.51	58.66	60.87	63.14	65.49
6.6	0.21	59.05	0.06	52.75	54.79	56.89	59.05	61.28	63.58	65.94
6.7	0.20	59.45	0.06	53.10	55.15	57.27	59.45	61.70	64.01	66.40
6.8	0.18	59.85	0.06	53.45	55.52	57.65	59.85	62.11	64.44	66.85
6.9	0.17	60.24	0.06	53.80	55.88	58.02	60.24	62.52	64.87	67.30
7	0.16	60.63	0.06	54.15	56.24	58.40	60.63	62.93	65.30	67.75
7.1	0.15	61.01	0.06	54.49	56.60	58.77	61.01	63.33	65.72	68.19
7.2	0.14	61.40	0.06	54.84	56.95	59.14	61.40	63.73	66.14	68.63
7.3	0.12	61.78	0.06	55.18	57.31	59.51	61.78	64.13	66.56	69.07
7.4	0.11	62.16	0.06	55.52	57.66	59.87	62.16	64.53	66.98	69.51
7.5	0.10	62.54	0.06	55.85	58.01	60.24	62.54	64.93	67.40	69.95
7.6	0.09	62.92	0.06	56.19	58.36	60.60	62.92	65.32	67.81	70.38
7.7	0.07	63.30	0.06	56.52	58.70	60.96	63.30	65.71	68.22	70.81
7.8	0.06	63.67	0.06	56.86	59.05	61.32	63.67	66.10	68.63	71.24
7.9	0.05	64.04	0.06	57.18	59.39	61.67	64.04	66.49	69.03	71.67
8	0.04	64.41	0.06	57.51	59.73	62.02	64.41	66.88	69.44	72.09
8.1	0.03	64.77	0.06	57.84	60.06	62.37	64.77	67.26	69.84	72.51
8.2	0.01	65.13	0.06	58.16	60.40	62.72	65.13	67.64	70.23	72.93
8.3	0.00	65.49	0.06	58.48	60.73	63.07	65.49	68.01	70.63	73.34
8.4	−0.01	65.85	0.06	58.80	61.06	63.41	65.85	68.39	71.02	73.76
8.5	−0.02	66.21	0.06	59.12	61.39	63.75	66.21	68.76	71.41	74.17
8.6	−0.03	66.56	0.06	59.43	61.71	64.09	66.56	69.13	71.80	74.57
8.7	−0.04	66.91	0.06	59.74	62.04	64.42	66.91	69.49	72.18	74.97
8.8	−0.05	67.25	0.06	60.05	62.36	64.76	67.25	69.85	72.56	75.37
8.9	−0.06	67.60	0.06	60.35	62.67	65.09	67.60	70.21	72.94	75.77
9	−0.07	67.94	0.06	60.66	62.99	65.41	67.94	70.57	73.31	76.16
9.1	−0.08	68.27	0.06	60.96	63.30	65.73	68.27	70.92	73.68	76.55
9.2	−0.09	68.61	0.06	61.25	63.60	66.05	68.61	71.27	74.04	76.93
9.3	−0.10	68.94	0.06	61.54	63.91	66.37	68.94	71.61	74.40	77.31
9.4	−0.11	69.26	0.06	61.83	64.21	66.68	69.26	71.95	74.76	77.69
9.5	−0.11	69.58	0.06	62.12	64.50	66.99	69.58	72.29	75.11	78.06
9.6	−0.12	69.90	0.06	62.40	64.80	67.30	69.90	72.62	75.46	78.43
9.7	−0.13	70.22	0.06	62.68	65.09	67.60	70.22	72.95	75.81	78.79
9.8	−0.14	70.53	0.06	62.96	65.38	67.90	70.53	73.28	76.15	79.15
9.9	−0.14	70.84	0.06	63.23	65.66	68.19	70.84	73.60	76.48	79.50
10	−0.15	71.14	0.06	63.50	65.94	68.48	71.14	73.92	76.82	79.85
10.1	−0.15	71.44	0.06	63.77	66.21	68.77	71.44	74.23	77.14	80.19
10.2	−0.16	71.73	0.06	64.03	66.49	69.05	71.73	74.54	77.47	80.53
10.3	−0.17	72.03	0.06	64.29	66.75	69.33	72.03	74.84	77.79	80.87
10.4	−0.17	72.31	0.06	64.54	67.02	69.61	72.31	75.14	78.10	81.20
10.5	−0.18	72.59	0.06	64.79	67.28	69.88	72.59	75.44	78.41	81.52
10.6	−0.18	72.87	0.06	65.04	67.54	70.14	72.87	75.73	78.71	81.84
10.7	−0.18	73.15	0.06	65.28	67.79	70.41	73.15	76.01	79.01	82.15
10.8	−0.19	73.42	0.06	65.52	68.04	70.67	73.42	76.29	79.31	82.46
10.9	−0.19	73.68	0.06	65.76	68.28	70.92	73.68	76.57	79.60	82.77
11	−0.19	73.94	0.06	65.99	68.52	71.17	73.94	76.84	79.88	83.06
11.1	−0.20	74.19	0.06	66.21	68.75	71.41	74.19	77.11	80.16	83.35
11.2	−0.20	74.44	0.06	66.43	68.98	71.65	74.44	77.37	80.43	83.64
11.3	−0.20	74.69	0.06	66.65	69.21	71.89	74.69	77.62	80.70	83.92
11.4	−0.20	74.93	0.06	66.86	69.43	72.12	74.93	77.87	80.96	84.19
11.5	−0.20	75.16	0.06	67.07	69.64	72.34	75.16	78.12	81.21	84.46
11.6	−0.20	75.39	0.06	67.27	69.85	72.56	75.39	78.35	81.46	84.71
11.7	−0.20	75.61	0.06	67.47	70.06	72.77	75.61	78.58	81.70	84.97
11.8	−0.20	75.83	0.06	67.66	70.26	72.98	75.83	78.81	81.94	85.21
11.9	−0.20	76.04	0.06	67.84	70.45	73.18	76.04	79.03	82.16	85.45
12	−0.20	76.24	0.06	68.02	70.64	73.37	76.24	79.24	82.38	85.68
12.1	−0.19	76.44	0.06	68.19	70.82	73.56	76.44	79.45	82.60	85.90
12.2	−0.19	76.63	0.06	68.36	70.99	73.75	76.63	79.65	82.81	86.11
12.3	−0.19	76.81	0.06	68.52	71.16	73.92	76.81	79.84	83.00	86.32
12.4	−0.18	76.99	0.06	68.68	71.33	74.10	76.99	80.02	83.20	86.52
12.5	−0.18	77.16	0.06	68.83	71.48	74.26	77.16	80.20	83.38	86.71
12.6	−0.17	77.33	0.06	68.97	71.64	74.42	77.33	80.37	83.56	86.89
12.7	−0.16	77.49	0.06	69.11	71.78	74.57	77.49	80.54	83.73	87.07
12.8	−0.16	77.64	0.06	69.24	71.92	74.72	77.64	80.70	83.89	87.24
12.9	−0.15	77.79	0.06	69.37	72.05	74.86	77.79	80.85	84.05	87.40
13	−0.14	77.93	0.06	69.49	72.18	74.99	77.93	80.99	84.20	87.55
13.1	−0.13	78.06	0.06	69.60	72.30	75.12	78.06	81.13	84.34	87.69
13.2	−0.11	78.19	0.06	69.71	72.42	75.24	78.19	81.26	84.48	87.83
13.3	−0.10	78.31	0.06	69.82	72.53	75.36	78.31	81.39	84.60	87.96
13.4	−0.09	78.43	0.06	69.91	72.63	75.47	78.43	81.51	84.73	88.08
13.5	−0.07	78.54	0.06	70.00	72.73	75.57	78.54	81.62	84.84	88.20
13.6	−0.06	78.64	0.06	70.09	72.83	75.67	78.64	81.73	84.95	88.30
13.7	−0.04	78.74	0.06	70.17	72.91	75.77	78.74	81.83	85.05	88.40
13.8	−0.03	78.83	0.06	70.25	73.00	75.86	78.83	81.93	85.15	88.50
13.9	−0.01	78.92	0.06	70.32	73.08	75.94	78.92	82.02	85.24	88.59
14	0.01	79.01	0.06	70.38	73.15	76.02	79.01	82.11	85.32	88.67
14.1	0.03	79.09	0.06	70.45	73.22	76.10	79.09	82.19	85.40	88.75
14.2	0.05	79.16	0.06	70.50	73.28	76.17	79.16	82.26	85.48	88.82
14.3	0.08	79.23	0.06	70.56	73.34	76.23	79.23	82.33	85.55	88.88
14.4	0.10	79.29	0.06	70.60	73.40	76.30	79.29	82.40	85.61	88.94
14.5	0.12	79.36	0.06	70.65	73.45	76.35	79.36	82.46	85.67	88.99
14.6	0.15	79.41	0.06	70.69	73.50	76.41	79.41	82.52	85.73	89.04
14.7	0.17	79.47	0.06	70.72	73.54	76.46	79.47	82.57	85.78	89.08
14.8	0.20	79.52	0.06	70.75	73.58	76.50	79.52	82.62	85.82	89.12
14.9	0.23	79.56	0.06	70.78	73.62	76.55	79.56	82.67	85.86	89.16
15	0.26	79.60	0.06	70.81	73.65	76.59	79.60	82.71	85.90	89.19
15.1	0.29	79.64	0.06	70.83	73.68	76.62	79.64	82.75	85.94	89.21
15.2	0.31	79.68	0.06	70.85	73.71	76.66	79.68	82.78	85.97	89.24
15.3	0.34	79.71	0.06	70.86	73.74	76.69	79.71	82.82	86.00	89.26
15.4	0.37	79.74	0.06	70.88	73.76	76.71	79.74	82.84	86.02	89.27
15.5	0.41	79.77	0.06	70.89	73.78	76.74	79.77	82.87	86.04	89.29
15.6	0.44	79.80	0.06	70.89	73.79	76.76	79.80	82.90	86.06	89.30
15.7	0.47	79.82	0.06	70.90	73.81	76.78	79.82	82.92	86.08	89.30
15.8	0.50	79.84	0.06	70.90	73.82	76.80	79.84	82.94	86.09	89.31
15.9	0.53	79.86	0.06	70.90	73.83	76.82	79.86	82.95	86.10	89.31
16	0.57	79.87	0.06	70.90	73.84	76.83	79.87	82.97	86.11	89.31
16.1	0.60	79.89	0.06	70.89	73.85	76.84	79.89	82.98	86.12	89.31
16.2	0.64	79.90	0.06	70.89	73.85	76.85	79.90	82.99	86.13	89.30
16.3	0.67	79.91	0.06	70.88	73.85	76.86	79.91	83.00	86.13	89.30
16.4	0.70	79.92	0.06	70.87	73.86	76.87	79.92	83.01	86.13	89.29
16.5	0.74	79.93	0.06	70.87	73.86	76.88	79.93	83.02	86.13	89.28
16.6	0.77	79.94	0.06	70.85	73.86	76.88	79.94	83.02	86.13	89.27
16.7	0.81	79.95	0.06	70.84	73.85	76.89	79.95	83.03	86.13	89.26
16.8	0.84	79.95	0.06	70.83	73.85	76.89	79.95	83.03	86.13	89.25
16.9	0.88	79.96	0.06	70.82	73.85	76.90	79.96	83.04	86.13	89.23
17	0.91	79.96	0.06	70.80	73.85	76.90	79.96	83.04	86.12	89.22
17.1	0.95	79.97	0.06	70.79	73.84	76.90	79.97	83.04	86.12	89.20
17.2	0.98	79.97	0.06	70.77	73.84	76.90	79.97	83.04	86.12	89.19
17.3	1.02	79.97	0.06	70.75	73.83	76.90	79.97	83.04	86.11	89.17
17.4	1.06	79.98	0.06	70.74	73.82	76.90	79.98	83.04	86.10	89.16
17.5	1.09	79.98	0.06	70.72	73.82	76.90	79.98	83.04	86.10	89.14
17.6	1.13	79.98	0.06	70.70	73.81	76.90	79.98	83.04	86.09	89.13
17.7	1.16	79.98	0.06	70.69	73.81	76.90	79.98	83.04	86.09	89.11
17.8	1.20	79.99	0.06	70.67	73.80	76.90	79.99	83.04	86.08	89.09
17.9	1.23	79.99	0.06	70.65	73.79	76.91	79.99	83.04	86.07	89.08
18	1.24	79.99	0.06	70.65	73.79	76.91	79.99	83.04	86.07	89.07

## Declaration of Competing Interest

The authors declare that they have no known competing financial interests or personal relationships that could have appeared to influence the work reported in this paper.
